# Muscle fatigue, bioenergetic responses and metabolic economy during load‐ and velocity‐based maximal dynamic contractions in young and older adults

**DOI:** 10.14814/phy2.15876

**Published:** 2023-11-23

**Authors:** Liam F. Fitzgerald, Miles F. Bartlett, Jane A. Kent

**Affiliations:** ^1^ Muscle Physiology Laboratory, Department of Kinesiology University of Massachusetts Amherst Massachusetts USA

**Keywords:** acidosis, aging, glycolysis, inorganic phosphate, oxidative phosphorylation, sarcopenia

## Abstract

We evaluated whether task‐dependent, age‐related differences in muscle fatigue (contraction‐induced decline in normalized power) develop from differences in bioenergetics or metabolic economy (ME; mass‐normalized work/mM ATP). We used magnetic resonance spectroscopy to quantify intracellular metabolites in vastus lateralis muscle of 10 young and 10 older adults during two maximal‐effort, 4‐min isotonic (20% maximal torque) and isokinetic (120°s^−1^) contraction protocols. Fatigue, inorganic phosphate (Pi), and pH (*p* ≥ 0.213) differed by age during isotonic contractions. However, older had less fatigue (*p* ≤ 0.011) and metabolic perturbation (lower [Pi], greater pH; *p* ≤ 0.031) than young during isokinetic contractions. ME was lower in older than young during isotonic contractions (*p* ≤ 0.003), but not associated with fatigue in either protocol or group. Rather, fatigue during both tasks was linearly related to changes in [H^+^], in both groups. The slope of fatigue versus [H^+^] was 50% lower in older than young during isokinetic contractions (*p* ≤ 0.023), consistent with less fatigue in older during this protocol. Overall, regardless of age or task type, acidosis, but not ME, was the primary mechanism for fatigue in vivo. The source of the age‐related differences in contraction‐induced acidosis in vivo remains to be determined, as does the apparent task‐dependent difference in the sensitivity of muscle to [H^+^].

## 1 INTRODUCTION

Muscle fatigue has been defined as the reduced capacity of a muscle to produce torque or power following a period of contractile activity (Kent‐Braun et al., 2012). Because activities of daily living, such as climbing stairs or walking, require repeated dynamic contractions (Petrella et al., 2005), the ability to resist muscle fatigue is especially important for older adults in order to maintain their mobility and quality of life (Theou et al., 2008). Numerous studies have reported less fatigue in older (≥~65 years) compared with young adults (≤~40 years) in response to isometric contractions (Chan et al., 2000; Chung et al., 2007; Ditor & Hicks, 2000; Kent‐Braun et al., 2002; Lanza et al., 2007). Conversely, greater fatigue has been observed in older compared with young adults in response to high‐velocity contractions (Baudry et al., 2007; Callahan & Kent‐Braun, 2011; Dalton et al., 2012, 2015; McNeil & Rice, 2007). This latter observation has been made in studies using both isokinetic and isotonic dynamic contractions (Callahan & Kent‐Braun, 2011; Dalton et al., 2012, 2015; Sundberg, Kuplic, et al., 2018). Notably, fatigue is similar in young and older adults in response to contractions at slow or moderate velocities (e.g., ≤120°s^−1^ in the knee extensor muscles; Callahan et al., 2009; Callahan & Kent‐Braun, 2011; Dalton et al., 2012; Lindström et al., 1997).

The relative fatigue resistance of older compared with young adults during isometric contractions is related to the greater accumulation of fatigue‐inducing metabolites (Pi, H^+^) in young muscle under these conditions (Kent‐Braun et al., 2002; Lanza et al., 2005, 2007). This accumulation is consistent with a greater proportion of ATP produced via oxidative phosphorylation (ATP_OX_) in older muscle during voluntary contractions (Callahan et al., 2016; Lanza et al., 2005, 2007). Because of this relatively greater use of oxidative ATP production, inorganic phosphate (Pi), proton (H^+^), and diprotonated phosphate (H_2_PO_4_^−^), which are known to inhibit force and velocity at the level of the crossbridges (Debold et al., 2006, 2008; Nosek et al., 1987), accumulate to a lesser extent in older compared with young muscle (Kent‐Braun et al., 2002; Lanza et al., 2005). Indeed, a computational model examining the etiology of age‐related differences in muscle fatigue supported the notion that fatigue resistance during isometric contractions is due to a lower reliance on glycolytic ATP production (Callahan et al., 2016). However, the mechanisms responsible for age‐related differences in fatigue during dynamic contractions are less well established.

Consistent with the notion that [Pi] and [H^+^] cause fatigue within muscle, recent work has shown that greater fatigue in older compared with young muscle in response to 4 min of maximal velocity contractions was associated with lower pH and greater [Pi] in aged muscle (Sundberg et al., 2019). Presumably this greater metabolic perturbation in aged muscle was due to a greater reliance on non‐oxidative ATP production in old (Callahan et al., 2016), however, this has yet to be established during a dynamic fatigue protocol in vivo. Furthermore, we recently reported an age‐related deficit in metabolic economy (ME; mass‐normalized torque or power produced per mM ATP consumed) of skeletal muscle during 24 s of isokinetic and isotonic, but not isometric, contractions (Fitzgerald et al., 2021). The question of whether lower ME (calculated in vivo from the sum of ATP production by the creatine kinase reaction, glycolysis and oxidative phosphorylation) in older adults may play a role in age‐related differences in fatigue during dynamic contractions remains unanswered.

Inadequate neural drive during voluntary contractions can also contribute to fatigue. The question of whether a lack of complete voluntary activation contributes to age‐related differences in fatigue has been investigated. Using stimulated isometric contractions before and after dynamic contractions, we and others have shown that greater fatigue in older than young adults in the knee extensor and plantar flexor muscles was not due to age‐related deficits in voluntary activation (Dalton et al., 2010; Fitzgerald et al., 2020; Sundberg, Kuplic, et al., 2018). Collectively, these results suggest that age‐related differences in fatigue are due to mechanisms localized within the muscle.

Therefore, the purpose of this study was to determine the extent to which differences in intracellular bioenergetics or ME contribute to age‐related differences in muscle fatigue in vivo during dynamic contractions. Given the task‐specificity of fatigue (Christie et al., 2011; Enoka & Duchateau, 2008), we used two maximal dynamic contraction modes to elicit power in different ways: isotonic contractions that emphasize velocity under low loads, and isokinetic contractions that emphasize maximal torque production at a fixed velocity. Based on the available literature, we hypothesized that (1) fatigue would be greater in older compared with young adults in response to 4 min of isotonic contractions against a load equivalent to 20% maximal isometric torque (MVDC_20%_), (2) this fatigue would be accompanied by greater [Pi], lower pH, and greater reliance on non‐oxidative ATP synthesis in older than young muscle, and (3) ME would be lower in older than young muscle during isotonic contractions. Conversely, based upon previous work (Callahan et al., 2009), we expected (4) no age‐related differences in muscle fatigue, [Pi], pH, or ATP production in response to 4 min of maximal isokinetic contractions at 120°s^−1^ (MVDC_120_). We further hypothesized that (5) ME would be lower in older than young adults during the isokinetic contractions.

## 2 MATERIALS AND METHODS

### 2.1 Participants

Ten young (24–35 years; *n* = 6 males) and 10 older (66–80 years; *n* = 5 males) healthy adults were studied. Prior to enrollment, each participant gave their written informed consent, as approved by the University of Massachusetts Amherst Institutional Review Board (2017‐4208), and in accordance with the Declaration of Helsinki. Physician's approval to participate was obtained for all older adults. Participants were sedentary by self‐report, defined as completing no more than one 30‐min session of structured exercise per week. They also reported being healthy, as evaluated by a health‐history questionnaire, and were not taking any medications known to affect physical function or muscle fatigue (e.g., beta‐blockers, calcium channel blockers, sedatives). Participants with significant arthritis in their lower extremities, cardiovascular symptoms upon exertion, metal implants, or who were claustrophobic were excluded from the study prior to enrollment. All participants answered “no” to all questions on the Physical Activity Readiness Questionnaire (Thomas et al., 1992), indicating that it was safe to have them participate. Portions of these data have been published previously (Fitzgerald et al., 2021).

### 2.2 Experimental design

Participants reported to the lab for three visits. At visit 1, physical function was measured, and participants were familiarized with the isotonic and isokinetic contraction protocols to be completed at visits 2 and 3. At the beginning of visit 2, magnetic resonance (MR) images of the entire thigh were obtained. Participants then completed 1 of 2 fatigue protocols while supine inside the MR scanner. The second fatigue protocol was completed at visit 3, which was at least 3 days following visit 2. Prior to the fatigue protocol at either visit 2 or 3, participants completed a rest‐contraction‐recovery protocol to quantify in vivo muscle oxidative capacity. The order of the fatigue protocols and measurement of oxidative capacity were randomized between visits 2 and 3. This study was designed to evaluate the potential role of bioenergetics and ME on age‐related differences in muscle fatigue during two different tasks. Importantly, it was not designed to compare differences across the two contraction protocols.

### 2.3 Physical function and physical activity

Measures of mobility and physical activity were obtained in order to characterize the study groups. Mobility was measured using the advanced short physical performance battery (SPPB‐A; Simonsick et al., 2001), as described previously (Fitzgerald et al., 2021). At the end of visit 1, participants were asked to wear a uniaxial accelerometer (Actigraph GT3X, Pensacola, FL) for 7 days, as described elsewhere (Fitzgerald et al., 2021). Average daily activity counts and minutes of moderate‐to‐vigorous physical activity (MVPA) were determined using ActiLife v6.13 software (ActiGraph, Pensacola, FL) with established cutpoints (Freedson et al., 1998).

### 2.4 Familiarization

Participants were seated on a Biodex 4 dynamometer (Biodex System 4, Shirley, NY) with hip and knee angles of 135° and 110°, respectively, to mimic the position used for the contraction protocols in the MR scanner at visits 2 and 3. After securing the participants in this position, they completed 2–3 maximal voluntary isometric contractions (MVICs; 3–5 s each with 1 min rest between contractions) to determine their maximal torque (Fitzgerald et al., 2020). Next, participants completed two 40‐s trials of dynamic contractions (1 every 2 s) with their dominant leg to familiarize them with the contraction protocols to be performed at visits 2 and 3. Each contraction was cued by an auditory signal and all contractions were maximal and performed over a 30° range of motion (110–140°). Participants were instructed to “kick their leg out as hard and fast as possible” for all contractions and given strong verbal encouragement throughout the familiarization. Visual torque feedback was provided via a computer monitor positioned at the participant's eye level.

### 2.5 Contractile volume

At Visit 2, MR images of the entire thigh were acquired using the Skyra 3 Tesla, 70 cm bore MR scanner (Siemens Medical Systems, Erlangen, Germany), as described elsewhere (Fitzgerald et al., 2021). Briefly, a multi‐echo (6‐point), 2D gradient‐echo Dixon sequence and 18‐channel flex coil combined with a 32‐channel spine coil were used to acquire 6 mm thick slices (6 mm thick) along the length of the thigh. Water and fat images in which all four quadriceps muscles were visible were analyzed, and fat‐free muscle cross‐sectional area (CSA, cm^2^), and volume (cm^3^) were estimated. The same investigator analyzed all MR images.

### 2.6 Muscle oxidative capacity

Participants were randomly assigned to complete this measure at either visit 2 or 3, prior to the fatigue protocol. Each participant was positioned inside the MR scanner with a dual‐tuned ^31^P/^1^H circular surface probe (^31^P coil: 8 cm diameter, ^1^H coil: 10.5 cm diameter) fastened over the vastus lateralis of their dominant leg using a bandage and velcro straps, as described previously (Bartlett, Fitzgerald, Nagarajan, Hiroi, et al. 2020; Bartlett et al., 2021; Fitzgerald et al., 2021). The coil used allowed sampling of tissue to a depth of 4 cm and provides a volumetric measurement of the intracellular metabolites in that tissue. The participant's knee and ankle were strapped to the ergometer, and an additional strap fastened over the hips to prevent unwanted movement (Jaber et al., 2020). Scout images were obtained to ensure the correct placement of the surface coil and positioning within the isocenter of the MR scanner. The homogeneity of the magnetic field was then optimized by shimming on the proton signal.

Next, participants completed a series of warm‐up and familiarization contractions consisting of three 5‐s MVICs, and three sets of either six maximal voluntary isokinetic contractions at 120°s^−1^ or 6 maximal voluntary isotonic contractions against a load equivalent to 20% MVIC. Participants then completed 24 s of maximal isokinetic contractions (1 every 2 s; 120°s^−1^), followed by a 10‐min recovery period in order to obtain post‐contraction PCr recovery measures of muscle oxidative capacity (Arnold et al., 1984; Meyer, 1988). Following 15 dummy pulses, ^31^P spectra were acquired for 60 s before, during, and 10 min following this protocol, using a 0.1 ms hard pulse, nominal 60° flip angle, 4000 Hz bandwidth, 2048 complex points, 2 s repetition time and 0.15 ms echo time.

All free induction decays were analyzed using jMRUI v6.0beta, as described elsewhere (Fitzgerald et al., 2021). During recovery, spectra were averaged to achieve the following temporal resolutions: 4 s for the first 20 s, 8 s for the next 280 s, and 30 s for the remaining 5 min. Peaks corresponding to PME, Pi, PDE, PCr and ATP were fit using Lorentzian lineshapes. Concentrations of Pi and PCr were calculated assuming that [Pi] + [PCr] = 42.5 mM in resting muscle and that free creatine ([fCr]) at rest is equal to resting [Pi] (Meyer, 1989). [ATP] in the resting muscle was assumed to equal = 8.2 mM (Harris et al., 1974). Intracellular pH was calculated based on the chemical shift of Pi relative to PCr (Moon & Richards, 1973). Broadening and splitting of the Pi peak can occur due to distinct intracellular pH pools during contractions (Park et al., 1987). When Pi splitting was evident, multiple Pi peaks were fit and the pH corresponding to each Pi pool was calculated separately (based on the chemical shift of each Pi pool relative to PCr). The weighted average for pH was then determined, as described previously (Lanza et al., 2005).

The repletion of PCr during recovery was fit with a mono‐exponential function and the rate constant (*k*_PCr_) determined, as shown in Equation 1:

(1) $$\mathrm{PCr}\left(t\right)=∆\mathrm{PCr}\left(1-e^{-k_{\mathrm{PCr}}}\right)+{\mathrm{PCr}}_{\mathrm{ex}}$$

where ΔPCr is the amplitude of PCr recovery, and PCr_ex_ is [PCr] at the end of the contraction. The rate constant of PCr recovery, *k*_PCr_, reflects the capacity for oxidative phosphorylation under these conditions (Kemp & Radda, 1994).

### 2.7 Fatigue protocols

Participants completed two fatigue protocols on separate days; 1 isotonic protocol against a load equivalent to 20% MVIC, and 1 isokinetic protocol at 120°s^−1^. For both protocols, participants performed 1 maximal voluntary dynamic contraction every 2 s for 4 min (120 contractions total). All contractions were performed over a 30° range of motion (110–140°, with 180° indicating a straight leg). Participants received verbal instructions and encouragement, visual torque feedback, and an auditory cue for each contraction.

Position, torque, and velocity were acquired and analyzed as described previously (Fitzgerald et al., 2021). To account for intersubject differences in muscle size, fatigue is reported here as the decline in normalized power (average power per cm^3^ muscle), for both protocols. The range of motion was also recorded. Fatigue was calculated as:

(2) $$\frac{\left(\text{average over final}\;10\;s\right)}{\left(\text{average of highest}\;2\;\text{contractions during first}\;20\;s\right)}\times 100$$

### 2.8 ATP production

For the fatigue protocols, spectra were averaged to achieve 60 and 10 s resolution at rest and during the fatigue protocol, respectively. For the recovery period, spectra were averaged to achieve the same temporal resolution as for the oxidative capacity measurement described above. Representative spectra from 1 young and 1 older male at rest and the end of the isotonic (Figure S1a [young] and Figure S1b [older]) and isokinetic (Figure S1c [young] and Figure S1d [older]) are provided in the supplementary materials. Following the determination of [PCr], [Pi], [ATP], and pH, ATP synthesis rates by the creatine kinase reaction (ATP_CK_) and glycolysis (ATP_GLY_) were calculated for both fatigue protocols, as described previously (Fitzgerald et al., 2021). Briefly, ATP production from the creatine kinase reaction is captured by changes in PCr. Estimates of intracellular buffering and changes in pH and PCr and the proton stoichiometry of the CK reaction coupled with ATP hydrolysis were used to calculate glycolytic ATP production (i.e., where the fate of pyruvate is lactate).

Oxidative ATP synthesis was calculated as follows (Bartlett, Fitzgerald, Nagarajan, & Kent, 2020):

(3) $${\mathrm{ATP}}_{\mathrm{OX}}=\frac{\left[V_{\max24}+\left(∆V_{\max}\times ∆t\right)\right]}{\left[1+{\left(K_{m}÷X\right)}^{nH}\right]}$$

where *V*_max24_ is the *V*_max_ measured from the PCr recovery kinetics after the 24 s oxidative capacity contraction protocol, Δ*t* the change in time from 30 s to each consecutive time point where ATP_OX_ was calculated, Δ*V*_max_ the linearly‐scaled rate of change in *V*_max_ observed during the fatigue protocols, *K*_*m*_ is the concentration of ADP at ½*V*_max_, and *n* ^H^ is the Hill coefficient describing the sigmoidal, second‐order control, relationship between ATP_OX_ and changes in [ADP] (Bartlett, Fitzgerald, Nagarajan, Hiroi, et al., 2020). *V*_max_, *K*_*m*_, and *n* ^H^ were determined using a multi‐parametric analysis, as described previously (Bartlett, Fitzgerald, Nagarajan, & Kent, 2020). The rate of change in *V*_max_ was calculated as:

(4) $$∆V_{\max}=\frac{\left(V_{\max240}-V_{\max24}\right)}{216}$$

where *V*_max240_ and *V*_max24_ are the *V*_max_ values calculated from the PCr recovery kinetics at the end of the fatigue protocols and at the end of the oxidative capacity contraction protocol, respectively. The difference between these values was divided by the difference in the duration of the contraction protocols (i.e., 240 s–24 s = 216 s). Finally, when PCr recovery following the fatigue protocols exhibited a bi‐exponential pattern, PCr recovery and *k*_PCr_ were modeled using a bi‐exponential equation as described elsewhere (Bartlett, Fitzgerald, Nagarajan, Hiroi, et al. 2020).

### 2.9 Metabolic economy

The ATP cost of contraction (mM J^−1^) was calculated for every 10‐s period as the total ATP synthesis (i.e., ∑ATP_CK_, ATP_GLY_, and ATP_OX_) divided by the ∑ concentric power for all contractions in that period. For each fatigue protocol, ME (W cm^−3^.mM ATP^−1^) was calculated as the ∑ normalized concentric power divided by the total ATP synthesis for each 10 s period (Fitzgerald et al., 2021).

### 2.10 Statistical analyses

All data were checked for normality and homogeneity of variance prior to any statistical comparisons using the Kolmogorov–Smirnov test and Levene's statistic, respectively. Differences in group descriptive characteristics; muscle morphology; baseline torque, velocity and power; muscle oxidative capacity; and fatigue (% initial normalized torque and power for the isokinetic protocol; % initial normalized velocity and power for the isotonic protocol) were analyzed using independent *t*‐tests. To test our hypotheses, group differences during the fatigue trials were evaluated using two‐factor (group, time) repeated measures ANOVAs, as follows: (1) normalized torque, normalized velocity, normalized power, range of motion, and the duration of concentric contraction; (2) metabolites (Pi, pH, and ATP); and 3) ATP production (from ATP_CK_, ATP_GLY_, ATP_OX_), total ATP, ATP cost of contraction, and ME. Post‐hoc analyses were performed where appropriate using Šídák's correction for multiple comparisons. Associations between muscle fatigue and: [H^+^], [Pi], [H_2_PO_4_^−^], and ME were evaluated in young and older groups using linear regression, and differences in slopes were determined by *t*‐test. All statistical analyses were performed using GraphPad Prism version 9.1.2 for Windows (GraphPad Software, San Diego, CA) with an alpha level of 0.05. Data are reported as mean ± SD, with exact *p*‐values and 95% confidence intervals for differences in group means. We did not evaluate the effects of sex due to the small sample size.

## 3 RESULTS

### 3.1 Baseline characteristics

Group characteristics are reported in Table 1. There were no group differences in height, body mass or BMI. Additionally, groups were well matched for total daily physical activity and reasonably so for minutes of MVPA. We observed no difference in chair rise time or SPPB‐A between groups, indicating no significant deficit in physical function in this group of healthy older adults.

**TABLE 1:** Group characteristics.

	Young (*n* = 10)	Older (*n* = 10)	95% CI	*p*
Age (years)	27.5 ± 3.9	71.2 ± 5.0	—	—
Height (m)	1.69 ± 0.1	1.64 ± 0.11	−0.46, 0.15	0.286
Body mass (kg)	75.3 ± 14.0	67.8 ± 12.4	−4.91, 19.87	0.221
BMI (kg m^−2^)	26.0 ± 2.7	24.9 ± 2.2	−1.27, 3.45	0.344
PA (counts day^−1^/1000)	238.7 ± 102.0	238.6 ± 99.5	−94.65, 94.73	0.999
MVPA (min day^−1^)	38.3 ± 17.8	27.2 ± 23.3	−8.39, 30.55	0.247
Chair rise time (s)	16.1 ± 4.0	19.7 ± 5.2	−7.85, 0.81	0.105
SPPB‐A	2.81 ± 0.27	2.57 ± 0.26	−0.005, 0.49	0.054

*Note*: Values are mean ± SD.

Abbreviations: 95% CI, 95% confidence intervals for the difference between group means; BMI, body mass index; MVPA, moderate‐ to vigorous‐intensity physical activity; PA, physical activity; SPPB‐A, advanced short physical performance battery.

Baseline muscle characteristics are reported in Table 2. As expected, fat‐free muscle volume was lower in older compared with young adults. The lack of difference in the percentage of the femur length analyzed indicates that similar portions of the knee extensor muscles were sampled for the contractile volume measurement in both groups. Maximal isotonic and isokinetic power were lower in older compared with young adults; these age‐related differences in power remained when normalized to contractile volume, demonstrating lower maximal normalized power in older for both contraction modes. In vivo oxidative capacity of the knee extensor muscle was not different between young and older groups.

**TABLE 2:** Baseline muscle characteristics.

	Young (*n* = 10)	Older (*n* = 10)	95% CI	*p*
Contractile volume (cm^3^)	1125.6 ± 347.5	803.5 ± 228.7	45.7, 598.4	0.025
Femur length analyzed (%)	46.1 ± 4.9	45.0 ± 5.7	−3.9, 6.1	0.651
Isotonic power (W)	367.7 ± 135.4	178.1 ± 84.5	29.4, 96.8	0.002
Isokinetic power (W)	238.5 ± 96.5	133.5 ± 62.1	28.7, 181.3	0.010
Normalized isotonic power (W cm^−3^)	0.328 ± 0.075	0.219 ± 0.072	0.1, 0.2	0.004
Normalized isokinetic power (W cm^−3^)	0.218 ± 0.070	0.161 ± 0.040	0.1, 0.1	0.037
*k*_PCr_ (s^−1^)	0.021 ± 0.004	0.022 ± 0.004	−0.004, 0.003	0.632

*Note*: Values are mean ± SD.

Abbreviations: 95% CI, 95% confidence intervals for the difference between group means; *k*_PCr_, rate constant of PCr recovery.

### 3.2 Isotonic fatigue trial

#### 3.2.1 Contractile measures

**FIGURE 1:**
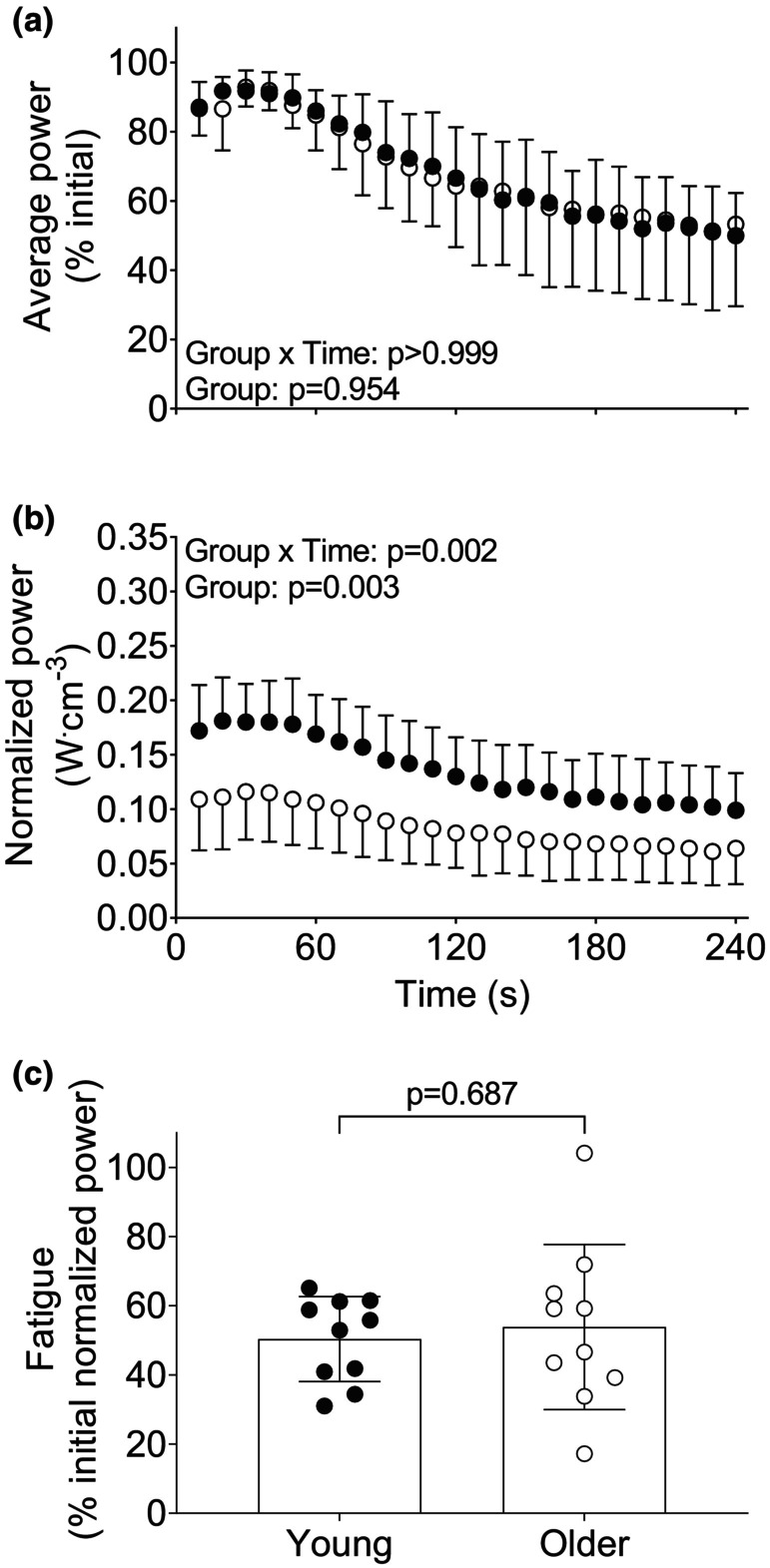
Changes in muscular performance in young and older groups during fatiguing isotonic contractions. A) average power as a percentage of initial power in young and older adults throughout the fatiguing isotonic contractions. B) average power normalized to muscle volume throughout the isotonic protocol. C) age‐related differences in muscle fatigue in young and older adults. Data are mean ± SD, with individual data points shown in panel C.

Contraction velocity averaged 176.3 ± 25.4°s^−1^ in the young group and 121.4 ± 36.7°s^−1^ in the older group over the first five contractions of the isotonic trial (*p* = 0.001). Changes in muscular performance during this fatigue protocol are shown in Figure 1. There were no main effects of group or group × time interactions for the changes in fatigue, expressed as a percentage of initial power (Figure 1a). There was a main effect of group and a group × time interaction for changes in normalized power during this protocol, such that the older had lower normalized power than the young at 50s and 60s of the fatigue protocol (*p* ≤ 0.046, Figure 1b). Additional data for average torque, peak acceleration, range of motion, concentric contraction duration, and average velocity during this protocol are shown in Figure S2. Overall, there were no differences in fatigue between groups during the velocity‐dependent isotonic contractions (Figure 1c), regardless of whether the age‐related difference in contractile volume was taken into account or not.

#### 3.2.2 Metabolites

**FIGURE 2:**
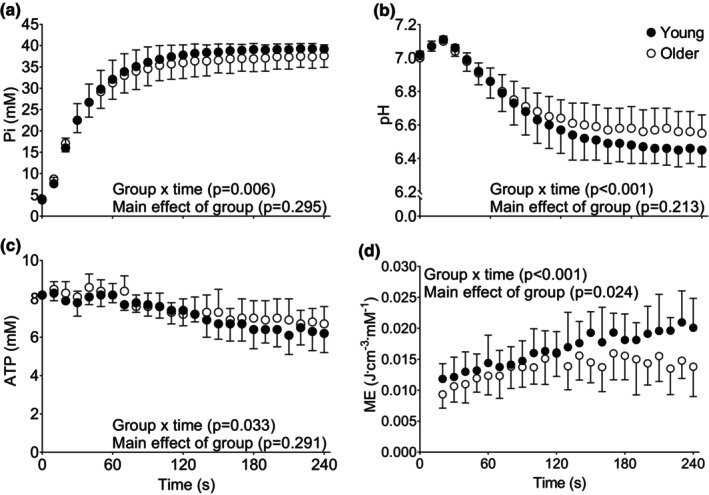
Changes in intracellular metabolites and metabolic economy (ME) during fatiguing isotonic contractions in young and older groups. Concentrations of Pi (A), H+ (B), ATP (C) are shown. Changes in metabolic economy are shown in panel D. Data are mean ± SD. ATP, adenosine triphosphate; Pi, inorganic phosphate.

Changes in [Pi], pH, and [ATP] during the 4 min of isotonic contractions are shown in Figure 2a–c. There were no main effects of group for [Pi], pH, or [ATP], but there were group × time interactions such that [Pi] was lower, and pH and [ATP] higher in the older overall, with no age‐related differences at any individual time point. Notably, [ATP] declined by ~20% in both groups by the end of the contractions (*p* ≤ 0.001, Figure 2c). Phosphocreatine declined to 8.5 ± 2.6% and 12.6 ± 7.0% of resting value in young and older muscle by the end of the isotonic contraction protocol, respectively (data not shown).

#### 3.2.3 ATP production

The production of ATP by the creatine kinase reaction, glycolysis, and oxidative phosphorylation during the 4 min of maximal isotonic contractions are shown in Figure S3a–c. There were no main effects of group and no group × time interactions for changes in ATP_CK_ or ATP_GLY_ during the 4 min of isotonic contractions. Similarly, there was no effect of group on changes in ATP_OX_, but there was a group × time interaction such that ATP_OX_ was lower towards the end of the protocol in young, but not older, muscle. However, post‐hoc analyses revealed no differences between groups at any specific time point.

#### 3.2.4 Metabolic economy

During the isotonic protocol, there was a main effect of group for ME such that ME was lower in older than in young (Figure 2d). Likewise, there was a significant group × time interaction such that ME continued to increase beyond ~120 s in the young, whereas ME plateaued in the older group during this period. Total ATP synthesis and the ATP cost of contraction, used to calculate ME, for the isotonic contractions are shown for the isotonic contractions in Figure S3d,e. There was no main effect of group on total ATP synthesis during this protocol, indicating that young and older muscle required similar ATP production (mM) despite the lower normalized power produced by the older muscle (Figure 1b). There was a group × time interaction such that ATP production in the older was lower during the first portion of the protocol compared with the young (Figure S3d), although the magnitude of these differences was modest. Finally, there was a main effect of group for the ATP cost of contraction (mM J^−1^), which was greater in older compared with young throughout the protocol (Figure S3e).

#### 3.2.5 Factors associated with isotonic fatigue

**FIGURE 3:**
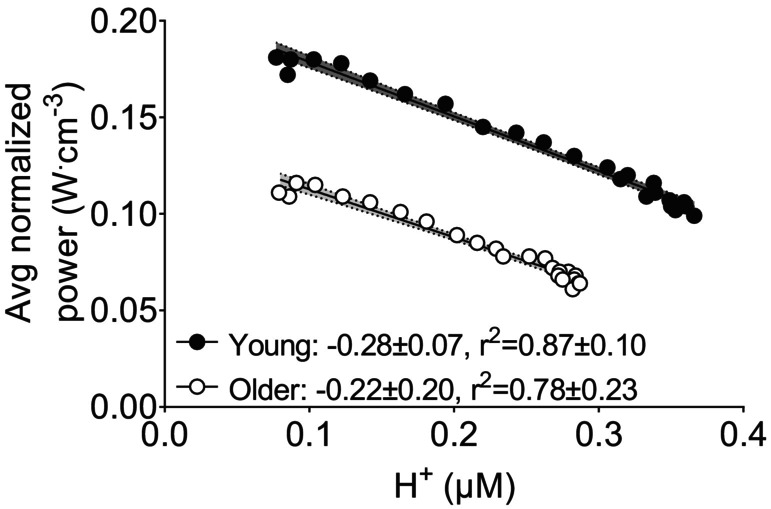
Associations between muscle fatigue and [H^+^] during fatiguing isotonic contractions in young and older groups. Data points are mean values with 95% confidence intervals. The mean ± SD slope and *r* ^2^ from the individual linear fits of the data are also shown. There was no difference between groups in the slope of the relationship between muscle fatigue and [H+] during the fatiguing isotonic contractions (*p* = 0.442).

As shown in Figure 3, changes in normalized power during the isotonic protocol were negatively associated with changes in [H^+^] in both groups. The mean of the individual slopes for these relationships was not different between groups (*p* = 0.442). Changes in normalized power were also negatively, but nonlinearly, associated with changes in [Pi] (*p* = 0.242) and [H_2_PO_4_^−^] (*p* = 0.239; Figure S4). Average ME during the 4 min of isotonic contractions was not associated with fatigue (%initial normalized power) in the young or older (*r* ^2^ = 0.182, *p* = 0.219 for both) groups (data not shown).

### 3.3 Isokinetic fatigue trial

#### 3.3.1 Contractile measures

**FIGURE 4:**
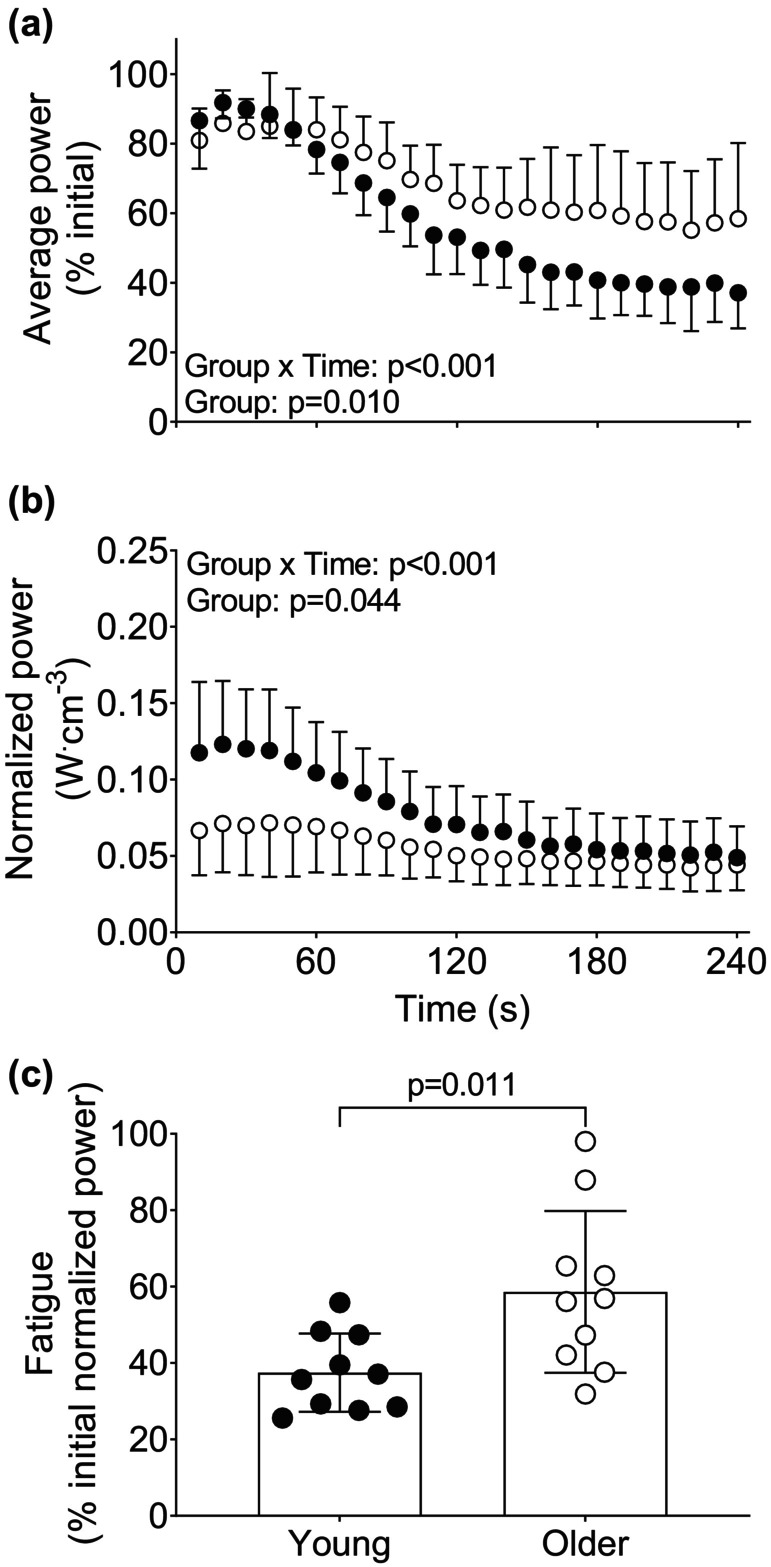
Changes in muscular performance in young and older groups during fatiguing isokinetic contractions. A) average power as a percentage of initial power in young and older adults throughout the fatiguing isokinetic contractions. B) average power normalized to muscle volume throughout the isokinetic protocol. C) age‐related differences in muscle fatigue in young and older adults. Data are mean ± SD, with individual data points shown in panel C.

Changes in muscular performance during the isokinetic fatigue protocol are shown in Figure 4. There were group × time interactions for average power as well as normalized power during this protocol (*p* < 0.001; Figure 4a,b). There were also main effects of group for average power and normalized power. Post‐hoc analyses indicated no group differences for normalized power at any individual time points. Muscle fatigue was greater in young than older whether differences in contractile volume were accounted for (Figure 4b) or not (Figure 4a). There was a main effect of group and a group × time interaction for changes in per cent initial torque, consistent with greater fatigue in young than older muscle (Figure S5e, *p* ≤ 0.002). There were no main effects of group or group × time interactions for the range of motion or duration of the concentric contraction during the isokinetic contraction protocol (Figure S5, *p* ≥ 0.182), although a main effect of group and group × time interaction indicated that peak acceleration was greater and decreased more in the young than the older during this protocol (Figure S5, *p* ≤ 0.010).

#### 3.3.2 Metabolites

**FIGURE 5:**
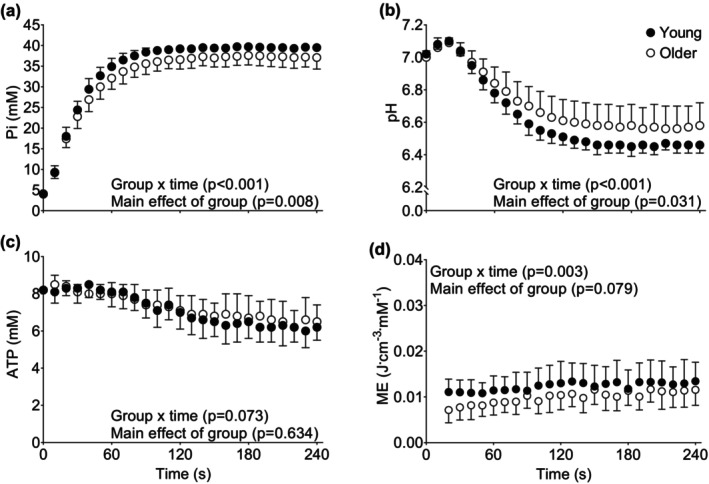
Changes in intracellular metabolites and metabolic economy (ME) in young and older groups during fatiguing isokinetic contractions. Concentrations of Pi (A), H+ (B), ATP (C) are shown. Changes in metabolic economy are shown in panel D. Supporting data in Figures S6d and S6e reveal that the ATP used per joule was greater in older than young muscle. Data are mean ± SD. ATP, adenosine triphosphate; Pi, inorganic phosphate.

Changes in [Pi], pH, and [ATP] throughout the 4 min of isokinetic contractions are shown in Figure 5a–c. There were main effects of group and group × time interactions for changes in [Pi] and pH such that Pi was lower and pH greater in the older compared with the young group. There was no main effect of group and no group × time interaction for changes in ATP during this protocol, although [ATP] declined by ~20% in both groups by the end of the 4 min (*p* ≤ 0.001, Figure 5c). Phosphocreatine declined to 7.7 ± 1.9% and 14.0 ± 7.1% of the resting value in young and older muscle by the end of the isokinetic contraction protocol, respectively (data not shown).

#### 3.3.3 ATP production

The production of ATP by the creatine kinase reaction, glycolysis, and oxidative phosphorylation during the 4 min of maximal isokinetic contractions is shown in Figure S6a–c. There were no main effects of group or group × time interactions for changes in ATP_CK_ (Figure S6a). In contrast, there was a main effect of group and a group × time interaction for changes in ATP_GLY_ during this protocol, such that ATP_GLY_ was lower in older than young, mainly during the first ~90 s of the protocol (Figure S6b); post‐hoc analyses revealed no differences between groups at any individual time point (*p* ≥ 0.255). Finally, there was no main effect of group for ATP_OX_, but there was a group × time interaction such that ATP_OX_ plateaued in the older group but declined somewhat in the young group in the final portion of the contraction protocol (Figure S6c).

#### 3.3.4 Metabolic economy

As shown in Figure 5d, there was a group × time interaction but no main effect of group for ME during the isokinetic protocol. In support of the ME results, there were group × time interactions (*p* < 0.001) for changes in total ATP synthesis and the ATP cost of contraction, and a main effect of group for ATP cost (*p* = 0.022), such that the ATP used per joule was greater in older than young muscle (Figure S6d,e).

#### 3.3.5 Factors associated with isokinetic fatigue

**FIGURE 6:**
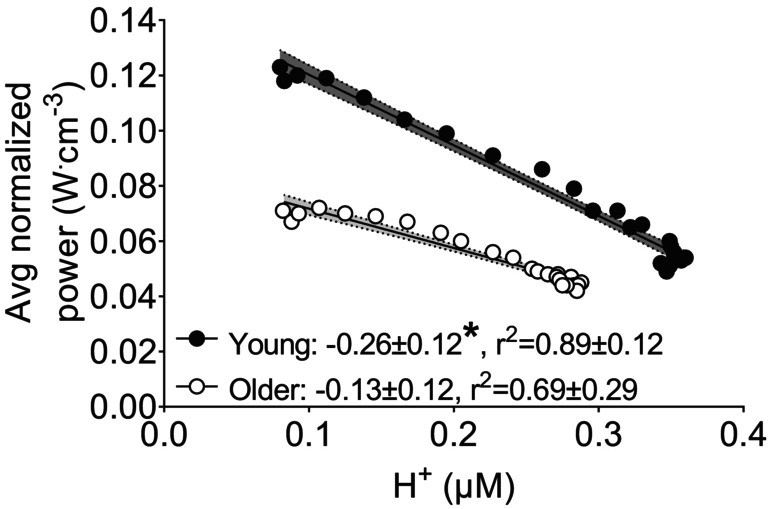
Associations between muscle fatigue and [H^+^] during fatiguing isokinetic contractions in young and older groups. Data points are mean values with 95% confidence intervals. The mean ± SD slope and *r* ^2^ from the individual linear fits of the data are also shown. Asterisk (*) indicates a difference in slopes between groups (*p* = 0.023).

As shown in Figure 6, changes in normalized power were negatively associated with changes in [H^+^] in both groups. The slope of the relationship between normalized power and [H^+^] was less steep in the older than young muscle (*p* = 0.023). Changes in normalized power were also nonlinearly associated with changes in [Pi] (*p* = 0.242) and [H_2_PO_4_^−^] (*p* = 0.239; Figure S7). Average ME during the isokinetic contraction protocol was not associated with fatigue (% initial normalized power) in either the young (*r* ^2^ = 0.004, *p* = 0.854) or older (*r* ^2^ = 0.032, *p* = 0.622) groups (data not shown).

## 4 DISCUSSION

This study was designed to evaluate whether intracellular bioenergetics (i.e., the pathway by which ATP is produced) or ME contribute to age‐related differences in muscle fatigue in vivo during two distinct bouts of maximal‐effort contractions of the knee extensor muscles. In contrast to our hypotheses for the velocity‐dependent isotonic contractions at 20% of MVIC, we found: (1) no difference in muscle fatigue between groups; (2) modestly lower [Pi] and greater pH in older than young muscle; and (3) lower ME in older that was not associated with fatigue. In response to the torque‐dependent isokinetic contractions at 120°s^−1^, we found: (4) less fatigue in older muscle that was accompanied by lower [Pi], greater pH and lower ATP_GLY_ in older than young muscle. As hypothesized, (5) ME was again lower in older than young muscle during isokinetic contractions, but was not associated with fatigue in either group. Rather, muscle fatigue in both protocols was linearly related to changes in [H^+^], regardless of age. Notably, the markedly lower slope in the older group for the relationship between changes in [H^+^] versus normalized power during isokinetic but not isotonic4 contractions suggests that the sensitivity of the contractile machinery to [H^+^] may change in old age in a task‐dependent manner (i.e., load‐ or velocity‐dependent contractions), potentially identifying a novel contributor to age‐related differences in muscle fatigue.

### 4.1 Baseline

By design, there were no differences in physical activity between groups in this study and all participants were relatively healthy based upon their BMI and mobility scores (Table 1). Muscle oxidative capacity was not different between age groups (*k*_PCr_, Table 2), which is in contrast with some previous work (Fitzgerald et al., 2016), but in agreement with others (Sundberg et al., 2019). The age of the groups in the current study is similar to those in previous studies that have observed an age‐related reduction in oxidative capacity of the knee extensor muscles (Larsen et al., 2012; Layec et al., 2015), suggesting the lack of difference observed in this study was not because of the age of the older group (i.e., old vs. very old). It has been shown that vastus lateralis muscle oxidative capacity is associated with average daily minutes of MVPA in young and older adults (Larsen et al., 2012) and, indeed, physical activity is a significant moderator of muscle oxidative capacity (*k*_PCr_) in aging (Fitzgerald et al., 2016). Thus, the lack of a difference in oxidative capacity between groups in the current study could be due to the relatively good health of the older adults, and the lack of difference in physical activity (both counts per day and minutes of MVPA) between our age groups. Because mitochondrial energetics are closely associated with maximal aerobic capacity and walking speed in older adults (Coen et al., 2013), future studies should elucidate the specific participant characteristics that are associated with lower muscle oxidative capacity with age.

As expected, contractile volume of the knee extensor muscles was lower in older than young adults (Table 2), which is consistent with previous reports (Callahan & Kent‐Braun, 2011; Hogrel et al., 2015). Peak isotonic and isokinetic power were lower in older than young adults both in absolute terms and when normalized to muscle volume, consistent with previous reports (Callahan & Kent‐Braun, 2011), indicating that the loss of peak power in aged muscle cannot be explained by muscle atrophy alone. Other factors that may contribute to a lower normalized power in unfatigued muscle of older adults include lower neural activation, changes in the molecular or cellular contractile function, or mechanical differences related to slower contractions over the smaller range of motion used in this study. However, others have found that the ability to fully recruit the knee extensors remains intact in older adults (Dalton et al., 2012; Sundberg, Kuplic, et al., 2018), suggesting that the lower normalized power in older adults might be due to changes in inherent muscle properties or increased co‐activation with age. While the source of this difference in normalized power is not clear at this time, the lower power production per unit muscle in the older group from the outset of the contraction protocols would likely have had an effect on the metabolic and thus fatigue responses during these contractions. That is, the energy demand for lower‐than‐expected power production would be lower as well, an interpretation that is supported by the smaller metabolic perturbations over time in the older group (Figure 2a–c).

### 4.2 Isotonic fatigue trial

Previous work has consistently shown greater fatigue in older than young adults in response to high‐velocity isokinetic (i.e., ≥240°s^−1^) or isotonic contractions (i.e., against a low‐load equivalent to ~20% MVIC) of the knee extensor muscles (Callahan & Kent‐Braun, 2011; Dalton et al., 2012, 2015; Fitzgerald et al., 2020; Sundberg, Kuplic, et al., 2018; Sundberg et al., 2019), as well as other muscles groups (Dalton et al., 2010; Wallace et al., 2016). A systematic review and meta‐analysis indicated greater muscle fatigue in older than young adults in studies where dynamic contractions were performed or muscular power was used as the index of fatigue (Christie et al., 2011). In contrast with these reports, here we observed no age‐related differences in muscle fatigue in response to 4 min of maximal‐effort isotonic contractions, regardless of whether fatigue was expressed as the per cent of initial normalized power, or power alone (Figure 1). In the current study, the average contraction velocity over the first 10 s of the isotonic fatigue protocol was ~120 and 180°s^−1^ in the older and young groups, respectively. Thus, one potential explanation for the lack of difference in muscle fatigue across age groups in our isotonic protocol is that the velocities achieved were too slow to elicit the age‐related differences in fatigue observed during high‐velocity contractions. Indeed, previous work has shown similar fatigue in older and young females when isokinetic contractions were performed at velocities corresponding to 75% maximal torque, which was ~78 and 104°s^−1^ in older and young, respectively (Callahan & Kent‐Braun, 2011). In addition to slower contraction velocities in our older group, the duration of each contraction was longer and the decline in the range of motion was greater in the older compared with the young adults (Figure S2). Each contraction was cued by a visual signal every 2 s, such that a longer contraction duration reflects an increase in the duty cycle. Thus, older adults fatigued similarly to the young despite a greater duty cycle and less rest between contractions. As shown in Figure S2c, the range of motion decreased to a greater extent in the older than young group, resulting in the older adults performing less work. This decrease in the range of motion and work in the older adults provides a potential explanation for the lack of difference in fatigue between groups, in contrast to that often observed during maximal‐effort, velocity‐dependent contractions.

We observed a group × time interaction for changes in [Pi], such that [Pi] was modestly lower in the older group throughout the isotonic contraction protocol (Figure 2). Moreover, pH was greater in older than young muscle by the end of the fatigue protocol. These observations are in contrast with a recent report evaluating age‐related differences in muscle fatigue and bioenergetics in response to 4 min of maximal voluntary contractions against a load equivalent to 20% MVIC (Sundberg et al., 2019). Specifically, those authors observed greater fatigue, [Pi] and [H_2_PO_4_^−^], and a lower pH in older than young muscle. Further, although we observed a ~20% decline in [ATP] in response to the isotonic contractions, Sundberg et al. reported no change in [ATP] in either group in their study. The source of the discrepancies between studies is not clear at present, but at least part of these differences in results may be due to the position of the participants inside the MR scanner and the different recruitment patterns of the knee extensor muscles in those positions. Participants in our study were supine and performed contractions over a 30° range of motion (Jaber et al., 2020). In contrast, participants in the Sundberg et al. study were prone, which can be an uncomfortable position from which to perform maximal knee extensions, and although the authors report an angular displacement of ~25–30°, the starting angle of the contractions was not reported. Thus, the participants may have been contracting over different portions of the force‐length relationship (Caldwell et al., 1993; Lanza et al., 2003).

Both our young and older groups showed an 18%–24% decline in [ATP] during the isotonic contraction protocol. In general, cytosolic [ATP] is well maintained during muscular contractions in vivo until [PCr] falls below ~20% of resting levels, such as during fatigue protocols or those designed to produce large metabolic perturbations (Taylor et al., 1986). For example, an early MRS study reported that, during dynamic forearm contractions, [ATP] decreased in participants who showed a greater decline in PCr (to 17 ± 5% of resting value) and pH (6.12 ± 0.04) than those participants in which PCr fell to 26 ± 5% and pH to 6.37 ± 0.09; [ATP] was unchanged in this latter group (Taylor et al., 1986). Likewise, in a recent study of young adults using the same isokinetic protocol reported here (Bartlett, Fitzgerald, Nagarajan, Hiroi, et al. 2020), 4 min of fatiguing contractions lowered [PCr] to ~10% of resting and pH to 6.6, and [ATP] declined by about 12%. In the present study, PCr declined to ~13% and 9% of resting, and pH fell to ~6.6 and 6.4 in older and young muscles, respectively. Together, these data support the concept that [ATP] is well preserved until [PCr] falls below 20% of initial levels, beyond which a net loss of [ATP] can be expected.

We observed no age‐related differences in ATP production by the creatine kinase reaction or glycolysis during the isotonic contraction protocol. Overall, ATP_OX_ was greater in older compared with young muscle during the isotonic contraction protocol, consistent with previous studies of the dorsiflexor muscles involving stimulated (Tevald et al., 2010), submaximal (Christie et al., 2014), and maximal (Christie et al., 2014; Lanza et al., 2005) contractions, all of which showed a relatively greater reliance on oxidative energy production. That is, older muscle with similar oxidative capacity as young (*k*_PCr_, Table 2) generates more ATP via oxidative phosphorylation. Additional work is needed to determine why this age‐related difference in bioenergetic pathway use exists in response to isometric and dynamic contractions.

### 4.3 Isokinetic fatigue trial

Older adults fatigued less than young in response to maximal‐effort isokinetic contractions at 120°s^−1^ (Figure 4), which is in agreement with a previous study that used dynamic contractions of the dorsiflexor muscles at 90°s^−1^ (Lanza et al., 2004). As observed with the isotonic contraction protocol, the lower normalized power in the early portion of the isokinetic fatigue protocol (Figure 4) indicates that the older group produced less than the expected work per unit muscle. Thus, young adults produced relatively greater torque during the isokinetic protocol compared with older adults, which may explain the greater fatigue in the young group.

In contrast with our hypotheses, we observed lower [Pi] and a greater pH in older than young muscle during the maximal isokinetic contractions (Figure 5). These results are consistent with previous studies of age‐related differences in fatigue in response to isometric contractions (Kent‐Braun et al., 2002; Lanza et al., 2005). The lower fatigue in older compared with young muscle in response to isometric contractions has been shown to be associated with a lower glycolytic ATP production and acidosis in older muscle (Callahan et al., 2016; Lanza et al., 2005). In the present study, we likewise found lower glycolytic ATP production in the older group during the isokinetic protocol (Figure S6). The underlying mechanism for a greater reliance on glycolytic ATP synthesis in young compared with older muscle is not clear at this time, but this difference does not appear to be due to an impairment in the glycolytic pathway with age because when blood flow is impeded with cuff‐induced ischemia, the peak glycolytic rate has been shown to be similar in older and young dorsiflexor muscles (Lanza et al., 2007). Overall, the smaller acidosis and increase in [Pi] in the older muscle was consistent with the development of less fatigue in this group.

As with the isotonic contraction protocol, during the isokinetic contractions, we observed a ~20% decline in [ATP] in our study groups. Once again, this was consistent with the fall of PCr to <15% of baseline concentrations during this fatigue protocol. Given that the ATP cost of contraction did not increase during these maximal‐intensity, fatiguing contractions, it is not clear why [ATP] declined. Regardless, this is a consistent observation (i.e., drop in [ATP] when [PCr] falls to <20% of resting) in MRS studies of bioenergetics in vivo.

A notable observation in both fatigue protocols is the greater pH in older than young muscle at the end of the contraction protocol, which is consistent with several studies (Callahan et al., 2016; Kent‐Braun et al., 2002; Lanza et al., 2005; Layec et al., 2015). One potential explanation for this difference in acidosis is the fiber‐type composition of older and young muscles. A long‐held belief is that type II muscle fibers are preferentially impacted by the aging process, resulting in muscles with relatively greater type I fiber area compared with young. However, a relatively greater proportion of type I fibers would likely result in increased ME, which is inconsistent with our results. Additionally and in contrast to some cross‐sectional studies, a longitudinal study revealed a shift towards a greater proportion of type II muscle fibers with age (Frontera et al., 2000). Therefore, the greater acidosis observed in young compared with older muscle in the current study is likely not due to shifts in myosin heavy chain expression with age. Alternatively, lower lactate dehydrogenase activity in older compared with younger muscle has been reported (Kaczor et al., 2006; Larsson et al., 1978; Pastoris et al., 2000), which could result in lower glycolytic flux in aging muscle. The question of whether age‐related changes in lactate dehydrogenase activity or isoenzyme can fully explain the lower pH in young compared with older muscle remains to be determined.

### 4.4 Metabolic economy

We recently reported similar total ATP synthesis but greater ATP cost and lower ME in older compared with young muscle, using the same groups as studied here, during 24‐s isotonic and isokinetic contractions of the unfatigued knee extensor muscles (Fitzgerald et al., 2021). To our knowledge, the current study is the first to directly examine the relationship between ME in vivo and fatigue induced by dynamic contractions in the context of aging. Here, ME was again substantially lower in older than young over the 4‐min duration of both fatigue protocols (Figures 2 and 5). As with our earlier study, this difference was established from the outset of these fatigue protocols by a greater ATP cost of contraction in the older than in the young, which in turn can be traced to the markedly lower normalized power in the older group coupled with comparable ATP production by both groups.

Overall, despite the differences by age in ME during the contractions, ME was not associated with fatigue in response to either protocol in young or older adults. These results are important because they are the first to reveal that ME does not appear to be a direct determinant of muscle fatigue in vivo.

### 4.5 Energetic basis of fatigue

We observed significant negative linear relationships in both age groups between changes in [H^+^] and normalized power during the isotonic (Figure 3) and isokinetic contractions (Figure 6). Together, these analyses of the evolution of fatigue over time indicate that approximately 70%–90% of the decrease in power per unit muscle was explained by [H^+^]. In contrast, the associations between measures of fatigue and [Pi] and [H_2_PO_4_^−^] were not as strong and were clearly nonlinear; indeed, little change in contractile capacity was evident below approximately 30 mM [Pi] and 15–20 mM [H_2_PO_4_^−^] (Figures S4 and S7). While a non‐linear fit could be used to evaluate these relationships, the simplest explanation of a strong dependency of fatigue on [H^+^], as shown in Figures 3 and 6, is appealing and clear. Notably, the graphs of [Pi] in Figures S4 and S7 indicate a steady increase in [Pi] at a time when there is little change in normalized power, up to approximately 30–35 mM of Pi, at which point performance then falls rapidly.

The strong associations between metabolite accumulation and fatigue observed here are consistent with reports in healthy young adults (Cady et al., 1989; Kent‐Braun, 1999) and in studies of aging (Kent‐Braun et al., 2002). At the molecular level, elevated [H^+^] reduces contraction velocity due to prolonged attachment of myosin to actin as a result of slowed ADP release (Debold et al., 2008, 2012; Knuth et al., 2006; Woodward & Debold, 2018). In addition, elevated [H^+^] decreases the force‐generating capacity of muscle, potentially through a competitive inhibition of Ca^2+^ binding with troponin‐C, thereby reducing the number of attached crossbridges (Unger & Debold, 2019; Woodward & Debold, 2018). Although a recent study observed similar depressive effects of both high [Pi] and [H^+^] on skeletal muscle fiber force, velocity, and power production in young and older males (Sundberg, Hunter, et al., 2018), those experiments were conducted at saturating [Ca^2+^], which is non‐physiological during fatigue in vivo because high [Pi] decreases Ca^2+^ release from the sarcoplasmic reticulum (Westerblad & Allen, 1991).

One of the novel results of the present study is that the slope of the relationships between changes in normalized power and [H^+^], were less steep in the older than young muscle during isokinetic contractions, suggesting lower sensitivity of the contractile machinery to changes in [H^+^] in older muscle during high‐load contractions. In contrast, the slope of the relationship between changes in normalized power and [H^+^] was not different between groups during isotonic contractions, suggesting there is no age‐related difference in the sensitivity of the contractile machinery to changes in [H^+^] during low‐load contractions. The source of these differences is not clear at present, but may be related to cytosolic [Ca^2+^]. Nonetheless, these findings provide a novel bioenergetic basis for the task‐dependent manner (i.e., load‐ or velocity‐dependent contractions) in which muscle fatigue presents in old age.

### 4.6 Limitations and other considerations

We did not include a measure of voluntary activation in the current study, which could have provided useful insight as to the ability of participants to fully activate the knee extensor muscles while supine inside the MR scanner. This decision was based upon the weight of evidence in the literature indicating that deficits in voluntary activation do not likely contribute to age‐related differences in muscle fatigue (Dalton et al., 2010; Fitzgerald et al., 2020; Sundberg, Kuplic, et al., 2018). Thus, although we cannot rule out the presence of voluntary activation failure in the current study, this possibility seems unlikely given previous work in this area, as well as the robust associations between H^+^ and fatigue observed in the present study in both groups.

Although our groups were reasonably well matched by sex and habitual physical activity, larger study groups would have allowed for stratification of the older group based upon sarcopenic status, as well as greater generalization to the aging population. Of note, previous work has shown that, while 12 weeks of progressive resistance training improved maximal strength in mobility‐limited older adults, it did not change the magnitude of muscle fatigue in response to 4 min of maximal isokinetic contractions of the knee extensor muscles (Englund et al., 2018). These results highlight the importance of evaluating both muscle strength (maximal torque or power production) and muscle fatigue, which are distinct characteristics of muscle with potential differences in their mechanisms of age‐related dysfunction.

Rather than using a maximal 4‐min contraction protocol, some studies have used a design whereby participants continue to contract until power output declines to a fixed percentage (e.g., 50% baseline power). Although this approach to quantifying muscle fatigue has utility, it suffers from great variability in the time to task failure, potentially due to differences in volition between participants. Using this approach of reaching a pre‐set threshold of baseline strength may be best suited for studies where recovery from fatigue is of primary interest, as it ensures that all participants recover from the same degree of fatigue.

Because isotonic contractions do not constrain velocity, they more closely resemble natural movements than isokinetic contractions, and therefore may provide results more generalizable to daily activities (Paris et al., 2022). However, in order to probe potential age‐related differences in the task specificity of muscle fatigue, the effects of age during both the velocity‐dependence of isotonic contractions and the torque‐dependence of isotonic contractions were evaluated.

Lastly, because intersubject variability in the responses to aging can be significant, particularly beyond ~80 years of age, categorizing older adults into smaller sub‐groups based on factors of interest (e.g., sarcopenic status, fatigability) may provide additional insight. Indeed, some studies have begun to use this approach and have observed greater muscle fatigue in very old (~86 years) compared with old (~70 years) adults, although the precise mechanisms responsible for this increased fatigue with advanced age are still poorly understood (Sundberg, Kuplic, et al., 2018). Building on the work reported here to investigate sub‐groups of older adults will be important in the future as we work to understand how muscle fatigue may contribute to mobility dysfunction in aging.

## 5 CONCLUSIONS

The results of this study demonstrate the potent effect of acidosis on muscle fatigue during both velocity‐ and load‐dependent contractions in young and older adults in vivo. Moreover, our results clearly show that ME is not associated with fatigue in either young or older knee extensor muscles. Importantly, this novel result was not known prior to this study. Additionally, our results show that the accumulation of H^+^ is strongly associated with muscle fatigue during both isotonic and isokinetic contractions in both young and older adults. That is, the magnitude of fatigue is largely determined by H^+^ accumulation, regardless of age or contraction type. Our data also suggest that the sensitivity of the contractile machinery to [H^+^] differs in old age in a contraction‐type dependent manner. This latter result may provide a novel explanation for age‐related differences in muscle fatigue under various conditions. Additional work will be needed in order to provide answers as to the underlying mechanisms for the difference in metabolic responses with age (greater acidosis and Pi in young during isokinetic but not isotonic contractions), and for the difference in sensitivity to H^+^ accumulation by contraction type.

## AUTHOR CONTRIBUTIONS

Liam F. Fitzgerald and Jane A. Kent conceived the study design. Liam F. Fitzgerald and Miles F. Bartlett collected and analyzed the data; Jane A. Kent guided these analyses. Liam F. Fitzgerald, Miles F. Bartlett, and Jane A. Kent interpreted the results. Liam F. Fitzgerald wrote the first draft of the manuscript. All authors contributed to the editing of the manuscript for intellectual content. All authors approved the final version of the manuscript submitted for publication and agree to be accountable for all aspects of the work.

## CONFLICT OF INTEREST STATEMENT

None of the authors have any conflicts of interest to disclose.

## ETHICAL STATEMENT

We confirm that we have read the Journal's position on issues involved in ethical publication and affirm that this report is consistent with those guidelines.

## Supporting information


Figure S1. Representative 31P spectra from 1 young and 1 older participant. Representative spectra at rest and the end of isotonic contractions are shown from: A) young male and B) older male. Representative spectra at rest and the end of the isokinetic contractions are shown for the same participants in C) young male and D) older male.

Figure S2. Changes in torque, peak acceleration, range of motion, concentric contraction duration, and velocity for each group during the isotonic contraction protocol. Data are mean±SD.

Figure S3. Changes in ATP production by A) the creatine kinase reaction, B) glycolysis, and C) oxidative phosphorylation; as well as D) total ATP and E) ATP cost of contraction during the isotonic protocol in young and older groups. Data are mean±SD.

Figure S4. The development of fatigue was not linearly associated with inorganic phosphate (Pi) and diprotonated phosphate (H2PO4‐) in young and older muscle during maximal isotonic contractions in vivo. Data are mean±SD. The mean±SD for each subject’s slope and r2 from a linear fit are also shown.

Figure S5. Changes in velocity, peak acceleration, range of motion, concentric contraction duration, and torque in young and older groups during isokinetic protocol. Data are mean±SD.

Figure S6. Changes in ATP production by A) the creatine kinase reaction, B) glycolysis, and C) oxidative phosphorylation; as well as D) total ATP and E) ATP cost of contraction during the isotonic protocol in young and older groups. Data are mean±SD.

Figure S7. The development of fatigue was not linearly associated with inorganic phosphate (Pi) or diprotonated phosphate (H2PO4‐) in young or older muscle during maximal isokinetic contractions in vivo. Data are mean±SD. The mean±SD for each subject’s slope and r2 from a linear fit are also shown. * indicates p〈0.05 for difference in slope between young and older groups.

## Data Availability

The data supporting the findings of this study are available from the corresponding author upon request.
