# Supplementary figures and images for: Muscle fatigue, bioenergetic responses and metabolic economy during load‐ and velocity‐based maximal dynamic contractions in young and older adults
Source: Physiol Rep. 2023 Nov 23;11(22):e15876. doi: 10.14814/phy2.15876 (PMC10667588; doi:10.14814/phy2.15876)

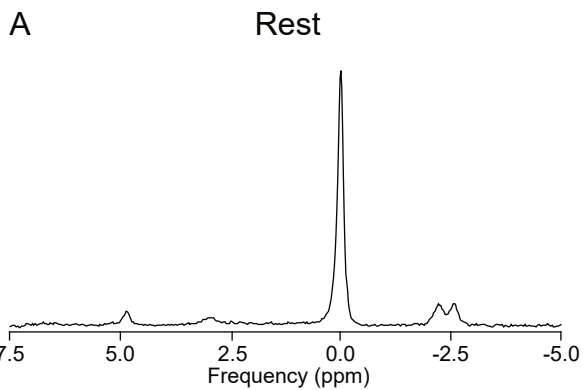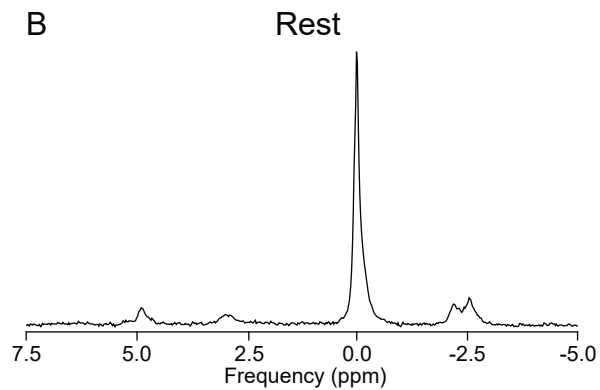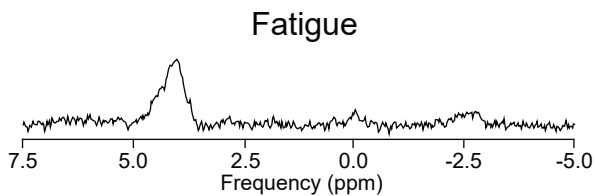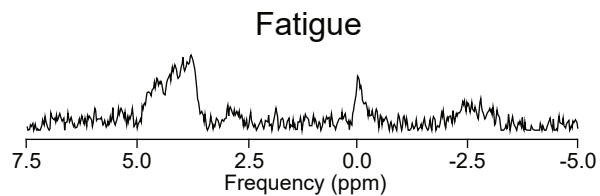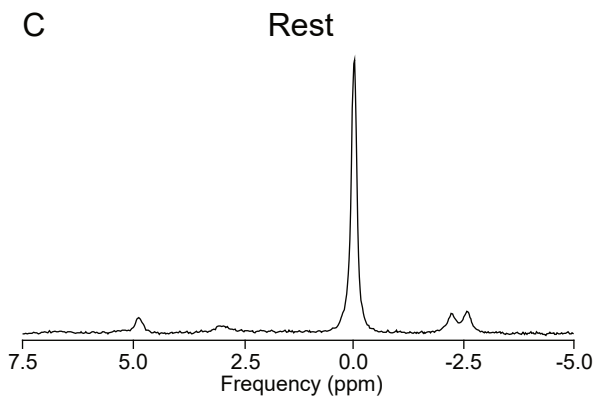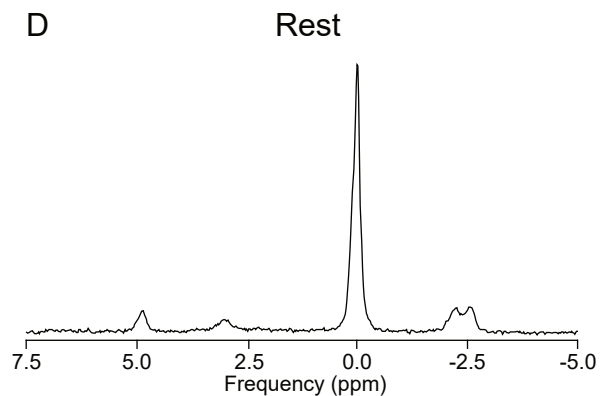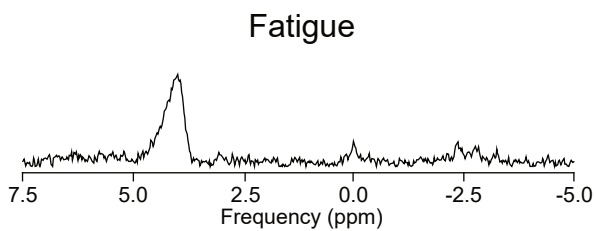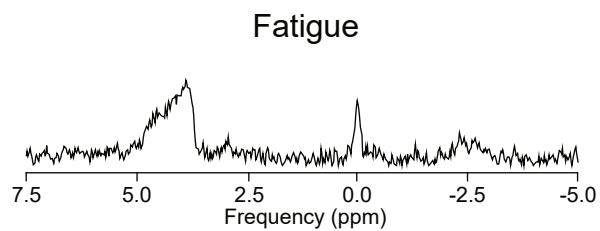

Supplement: Supplementary file 1 — Figure S1. Representative 31P spectra from 1 young and 1 older participant. Representative spectra at rest and the end of isotonic contractions are shown from: A) young male and B) older male. Representative spectra at rest and the end of the isokinetic contractions are shown for the same participants in C) young male and D) older male. [file PHY2-11-e15876-s006.pdf]

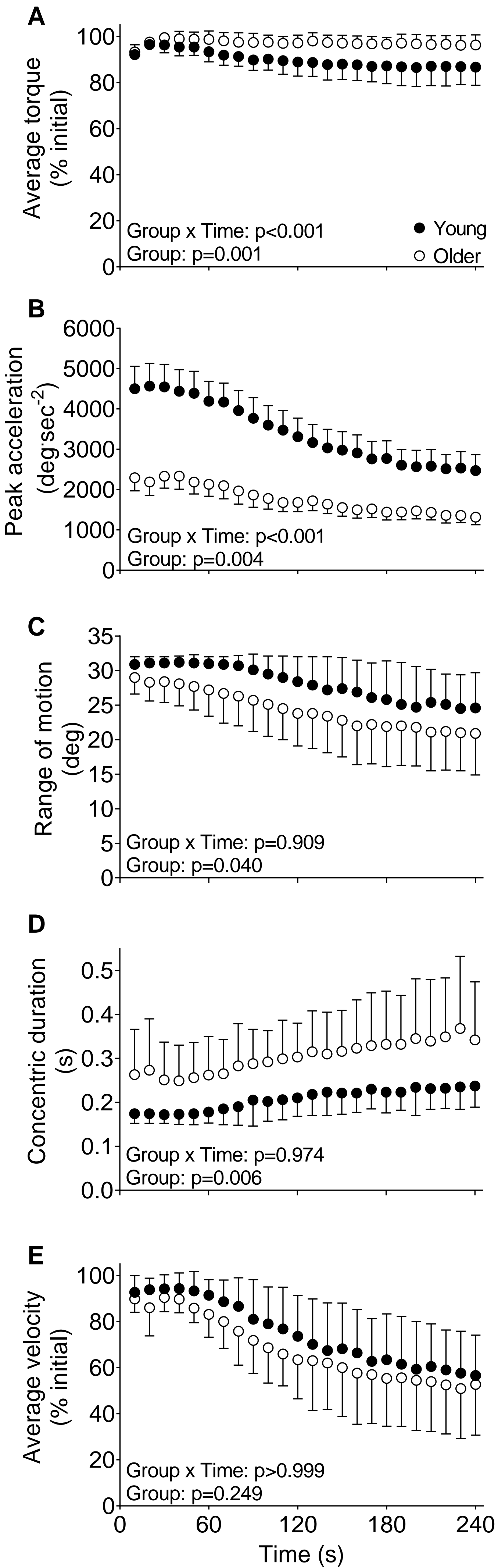

Supplement: Supplementary file 2 — Figure S2. Changes in torque, peak acceleration, range of motion, concentric contraction duration, and velocity for each group during the isotonic contraction protocol. Data are mean±SD. [file PHY2-11-e15876-s007.pdf]

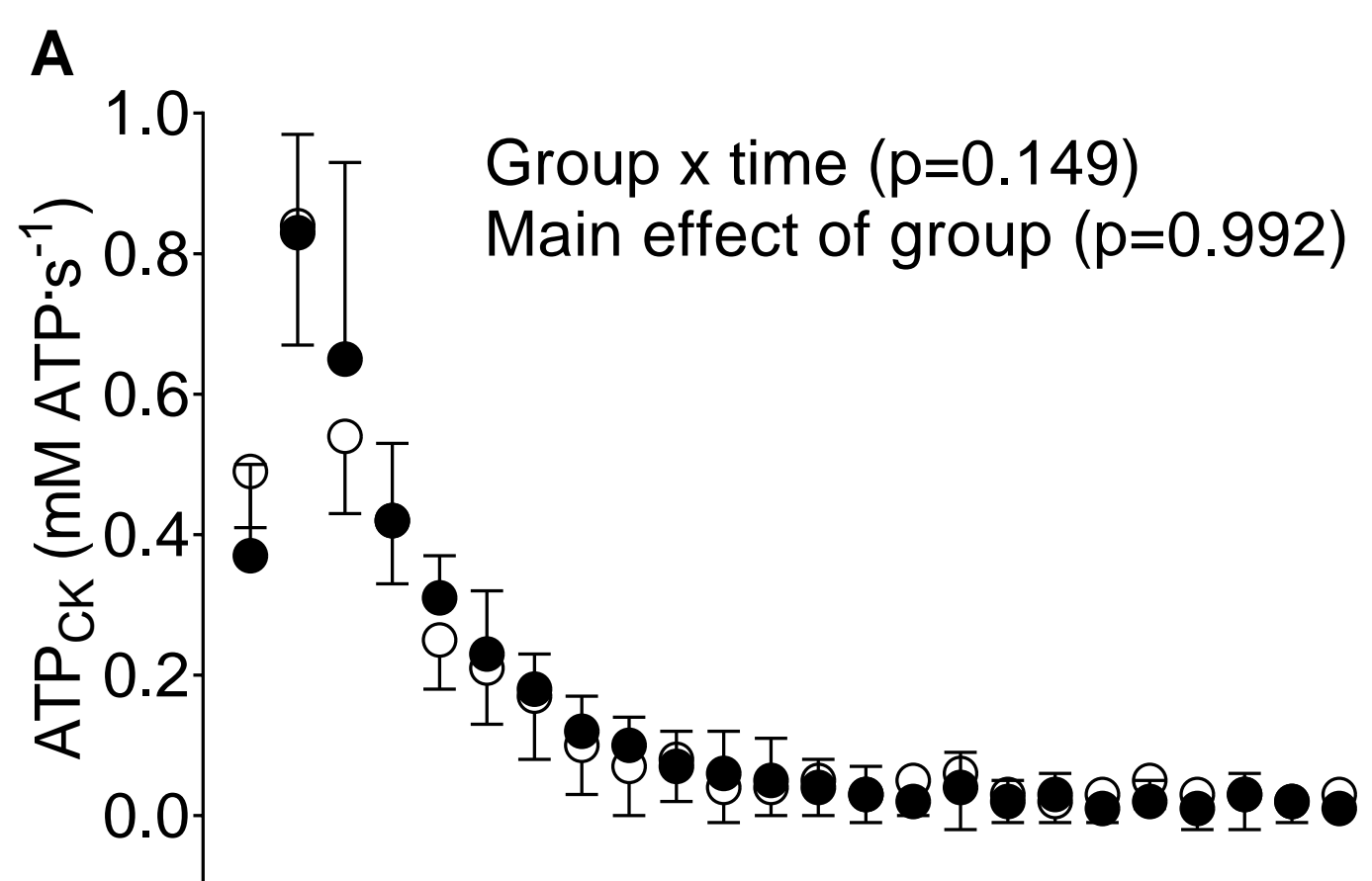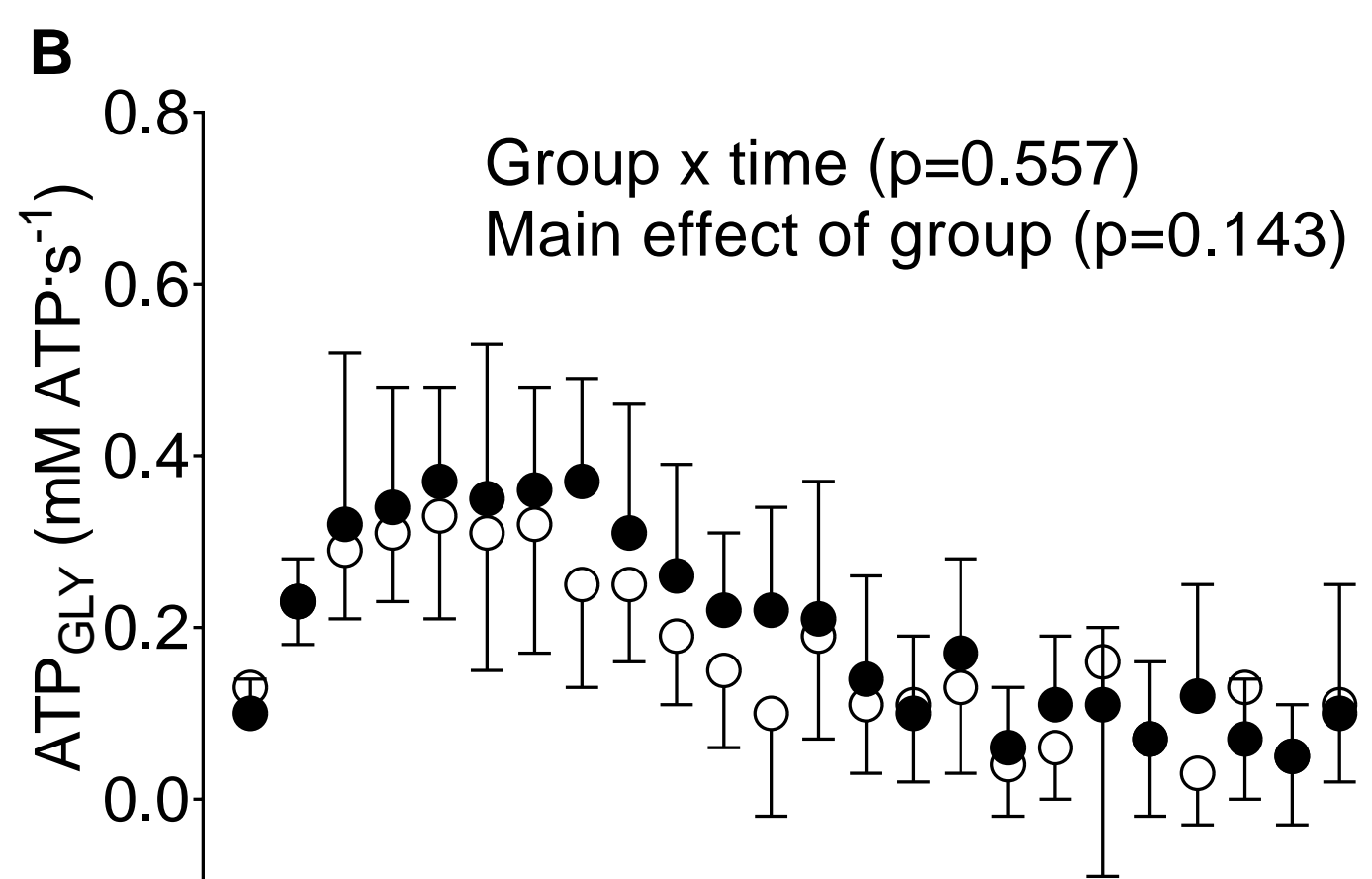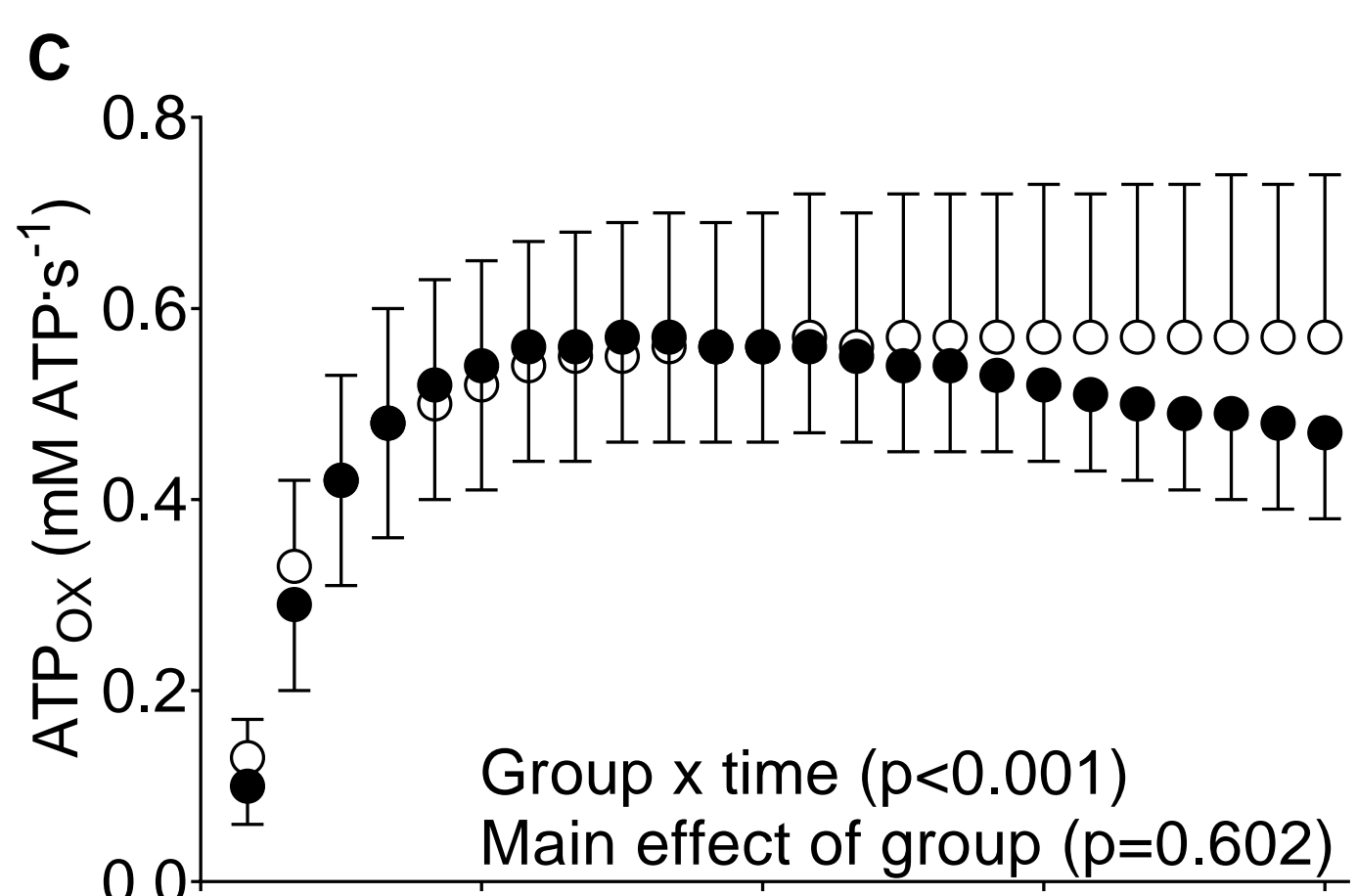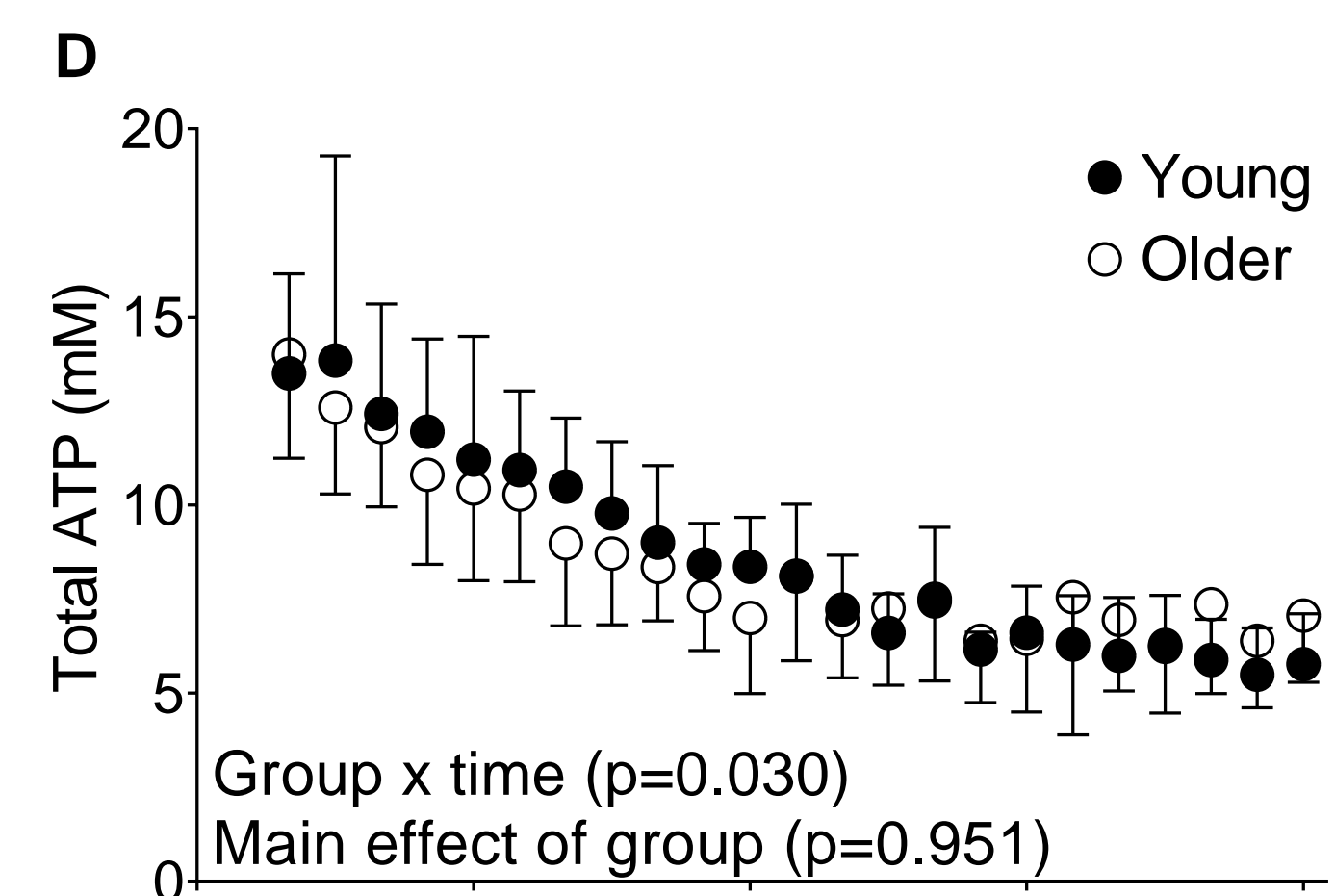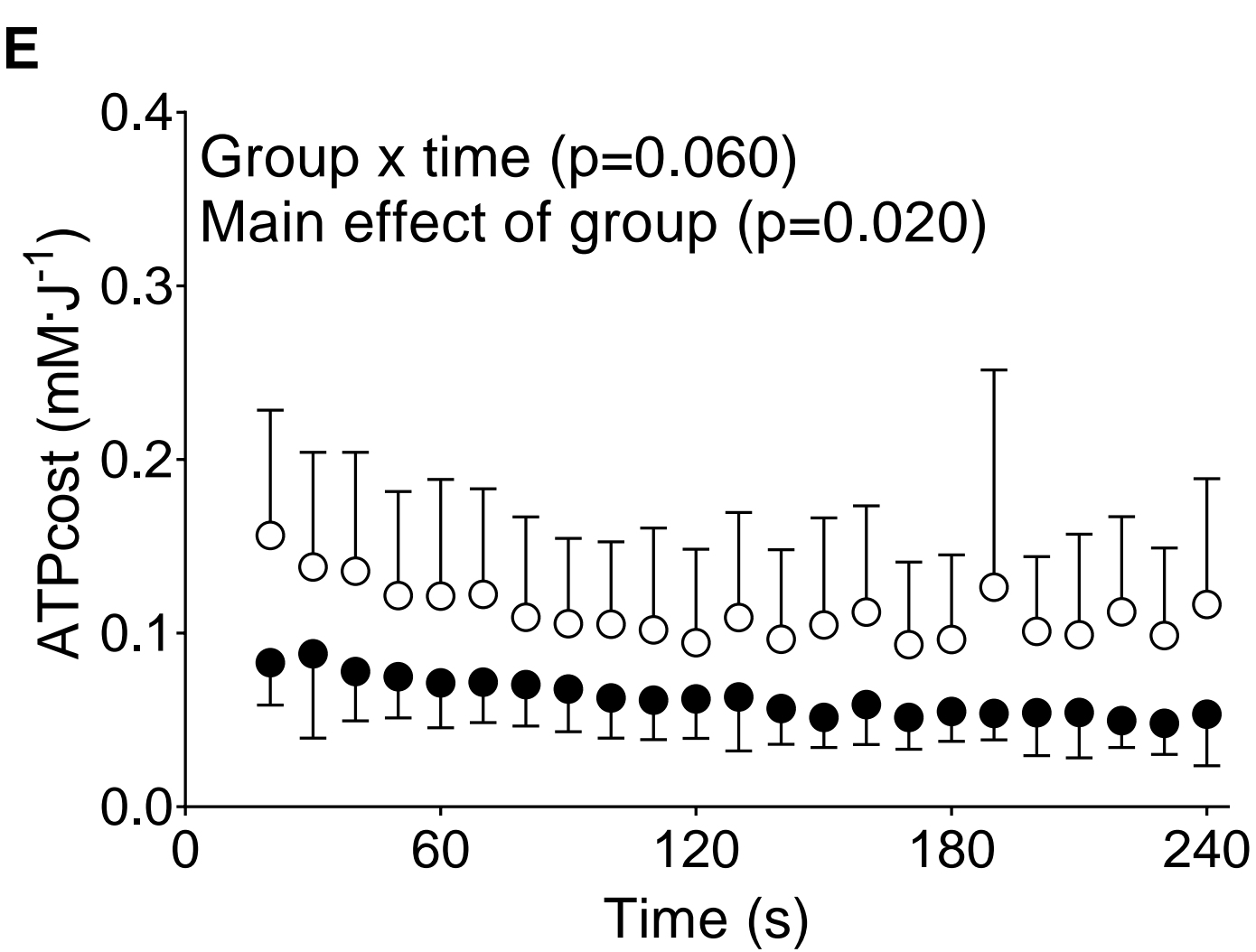

Supplement: Supplementary file 3 — Figure S3. Changes in ATP production by A) the creatine kinase reaction, B) glycolysis, and C) oxidative phosphorylation; as well as D) total ATP and E) ATP cost of contraction during the isotonic protocol in young and older groups. Data are mean±SD. [file PHY2-11-e15876-s005.pdf]

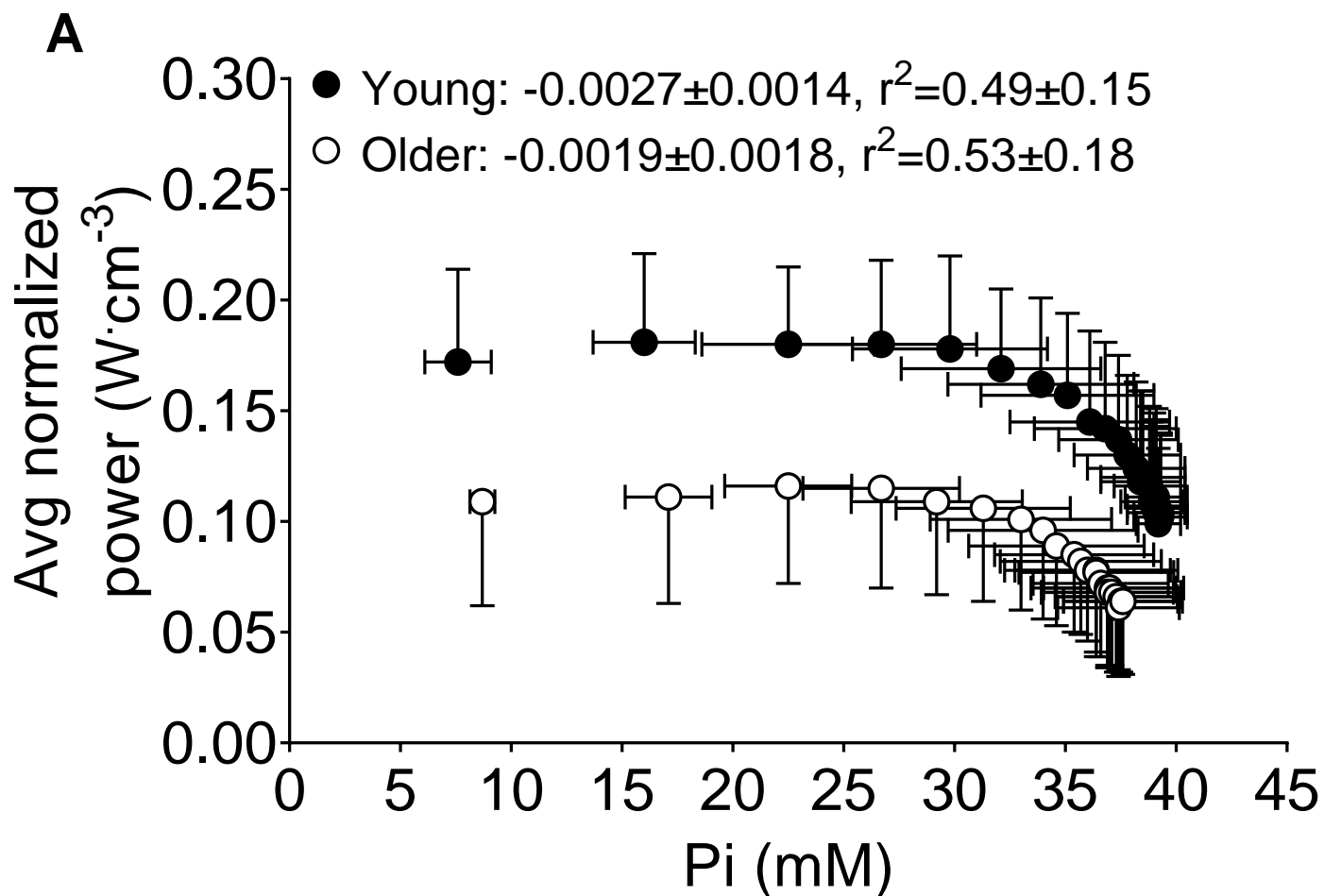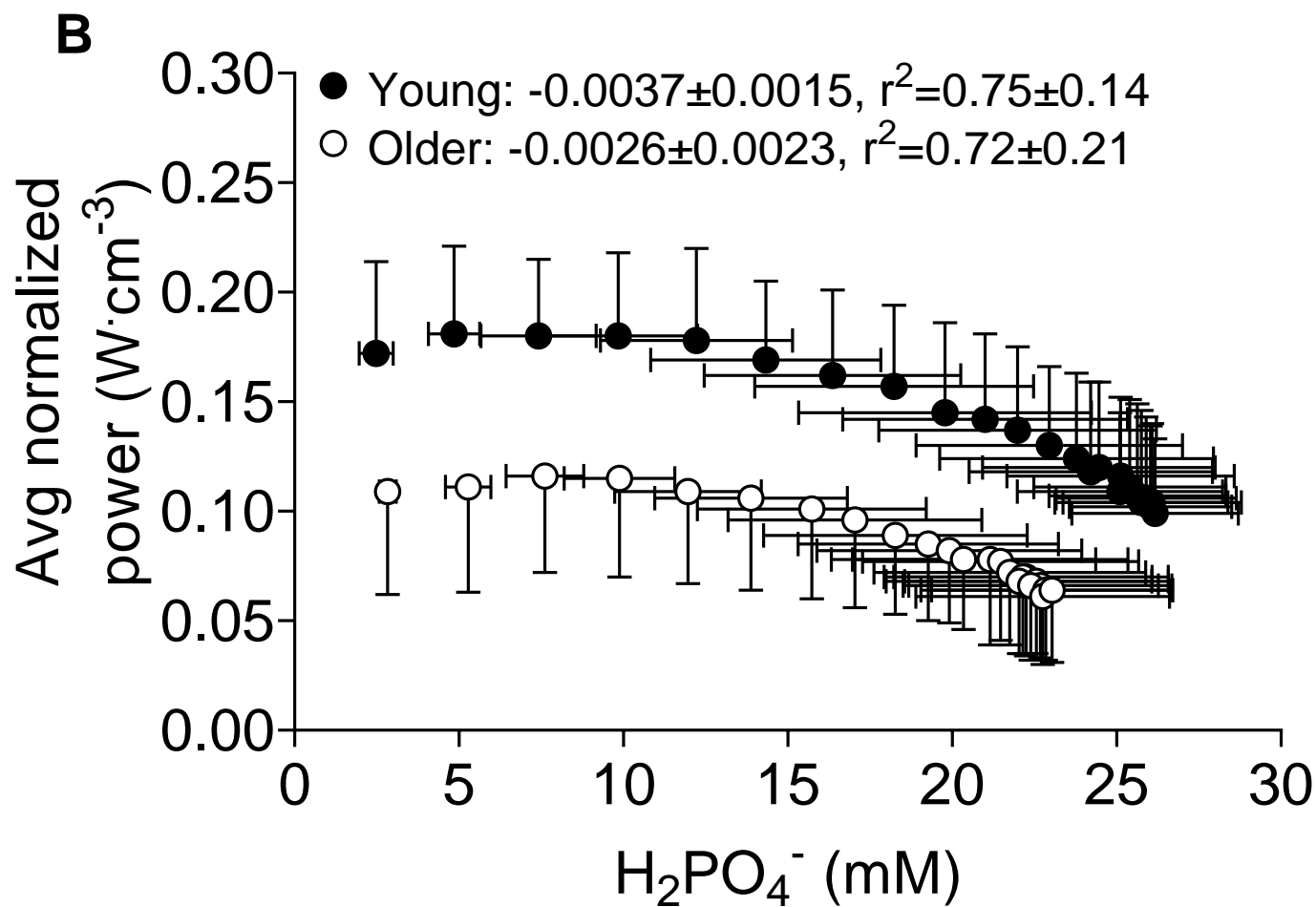

Supplement: Supplementary file 4 — Figure S4. The development of fatigue was not linearly associated with inorganic phosphate (Pi) and diprotonated phosphate (H2PO4‐) in young and older muscle during maximal isotonic contractions in vivo. Data are mean±SD. The mean±SD for each subject’s slope and r2 from a linear fit are also shown. [file PHY2-11-e15876-s004.pdf]

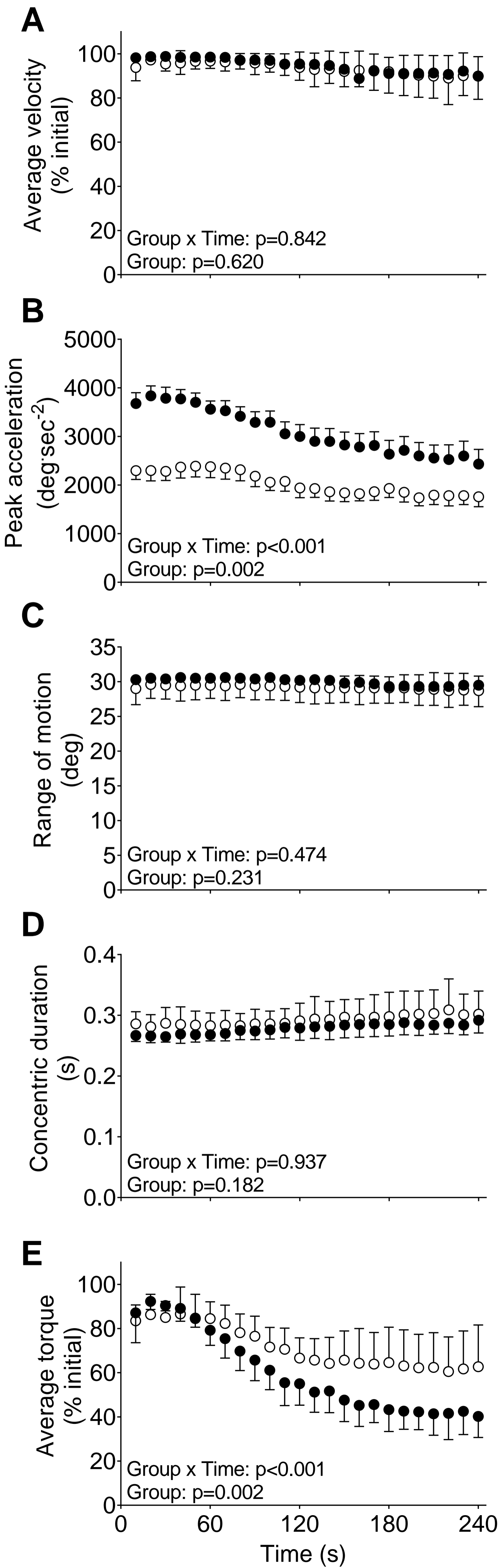

Supplement: Supplementary file 5 — Figure S5. Changes in velocity, peak acceleration, range of motion, concentric contraction duration, and torque in young and older groups during isokinetic protocol. Data are mean±SD. [file PHY2-11-e15876-s003.pdf]

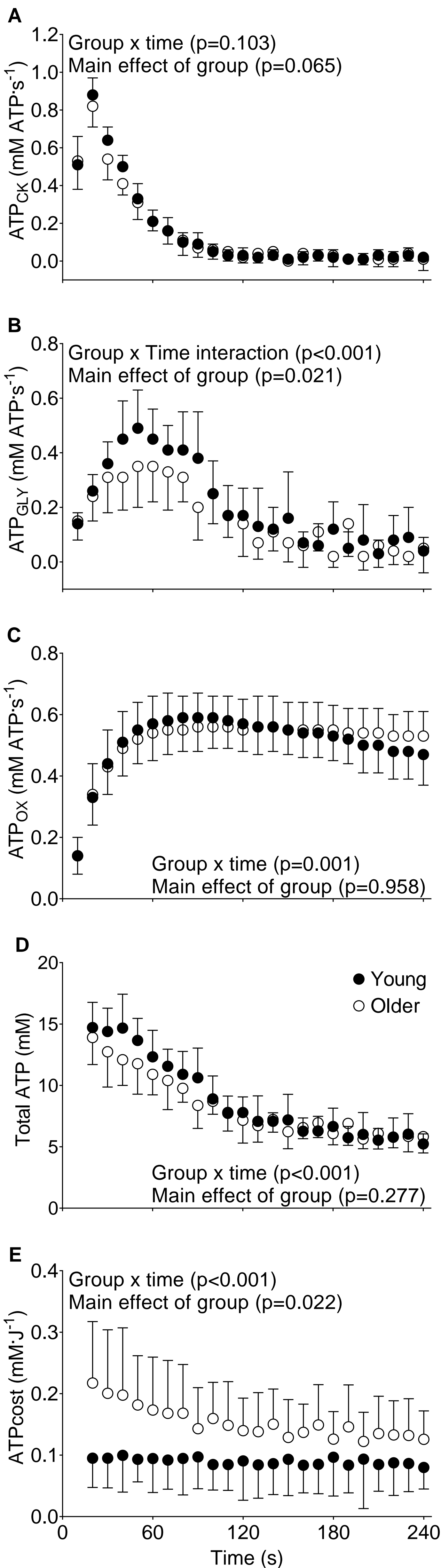

Supplement: Supplementary file 6 — Figure S6. Changes in ATP production by A) the creatine kinase reaction, B) glycolysis, and C) oxidative phosphorylation; as well as D) total ATP and E) ATP cost of contraction during the isotonic protocol in young and older groups. Data are mean±SD. [file PHY2-11-e15876-s002.pdf]

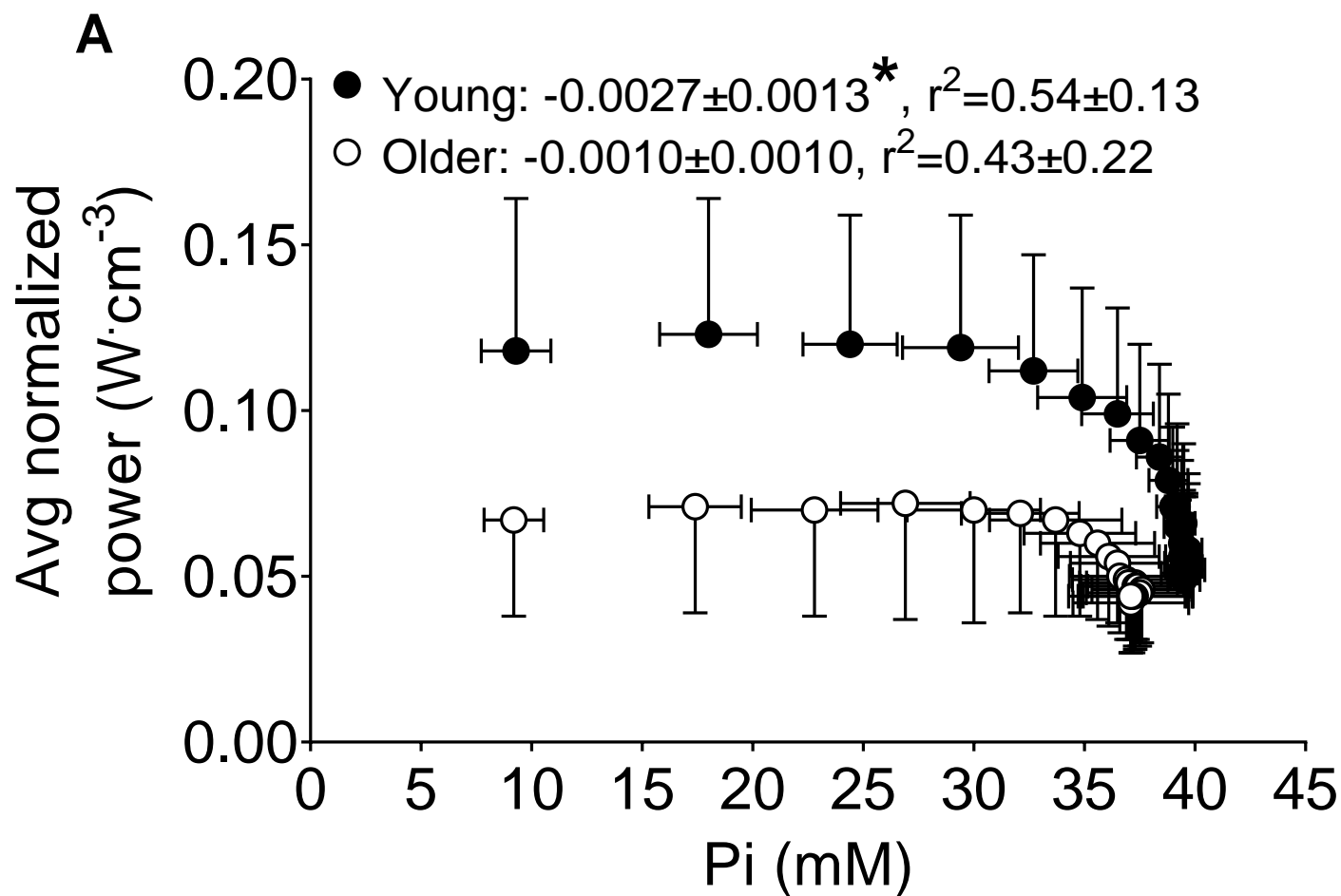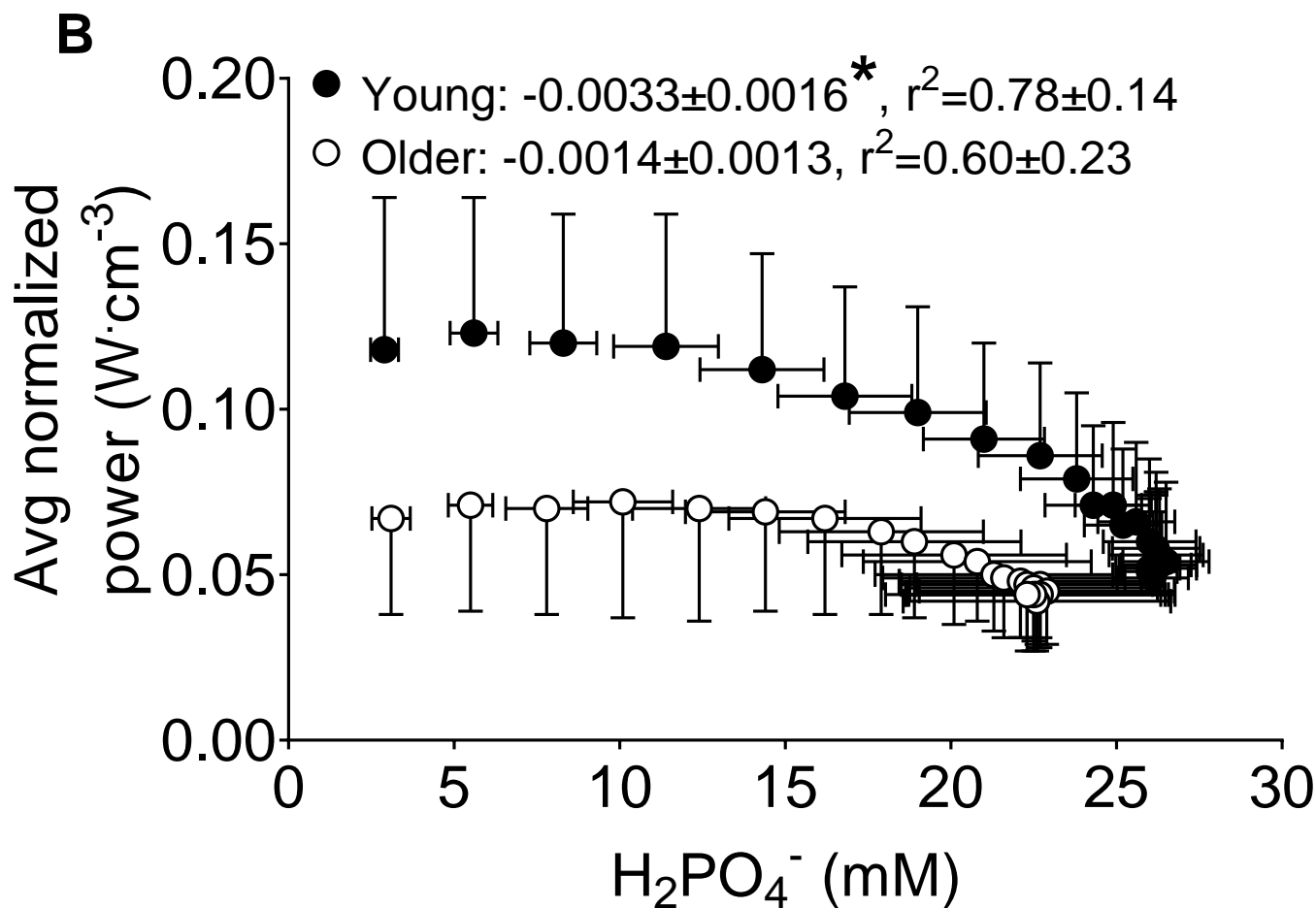

Supplement: Supplementary file 7 — Figure S7. The development of fatigue was not linearly associated with inorganic phosphate (Pi) or diprotonated phosphate (H2PO4‐) in young or older muscle during maximal isokinetic contractions in vivo. Data are mean±SD. The mean±SD for each subject’s slope and r2 from a linear fit are also shown. * indicates p〈0.05 for difference in slope between young and older groups. [file PHY2-11-e15876-s001.pdf]
